# Adiposity and infection-related mortality: Mendelian randomization study of 126 000 Mexican adults

**DOI:** 10.1093/ije/dyag197

**Published:** 2026-09-11

**Authors:** Lukman Lawal, Eirini Trichia, Jesus Alegre-Díaz, Paulina Baca, Elizabeth Barerra, Carlos González-Carballo, Raúl Ramirez-Reyes, Fernando Rivas, Diego Aguilar-Ramirez, Fiona Bragg, William G Herrington, Michael Hill, Tianshu Liu, Michael Turner, Jason Torres, Alejandra Vergara-Lope, Rachel Wade, Rory Collins, Pablo Kuri-Morales, Jaime Berumen, Jonathan R Emberson, Roberto Tapia-Conyer, Louisa Gnatiuc Friedrichs

**Affiliations:** Clinical Trial Service Unit & Epidemiological Studies Unit, Nuffield Department of Population Health, University of Oxford, Oxford, United Kingdom; Clinical Trial Service Unit & Epidemiological Studies Unit, Nuffield Department of Population Health, University of Oxford, Oxford, United Kingdom; Experimental Research Unit from the Faculty of Medicine, National Autonomous University of Mexico, Mexico City, Mexico; Experimental Research Unit from the Faculty of Medicine, National Autonomous University of Mexico, Mexico City, Mexico; Experimental Research Unit from the Faculty of Medicine, National Autonomous University of Mexico, Mexico City, Mexico; Experimental Research Unit from the Faculty of Medicine, National Autonomous University of Mexico, Mexico City, Mexico; Experimental Research Unit from the Faculty of Medicine, National Autonomous University of Mexico, Mexico City, Mexico; Experimental Research Unit from the Faculty of Medicine, National Autonomous University of Mexico, Mexico City, Mexico; Clinical Trial Service Unit & Epidemiological Studies Unit, Nuffield Department of Population Health, University of Oxford, Oxford, United Kingdom; Clinical Trial Service Unit & Epidemiological Studies Unit, Nuffield Department of Population Health, University of Oxford, Oxford, United Kingdom; Health Data Research UK Oxford, University of Oxford, Oxford, United Kingdom; Clinical Trial Service Unit & Epidemiological Studies Unit, Nuffield Department of Population Health, University of Oxford, Oxford, United Kingdom; Clinical Trial Service Unit & Epidemiological Studies Unit, Nuffield Department of Population Health, University of Oxford, Oxford, United Kingdom; Clinical Trial Service Unit & Epidemiological Studies Unit, Nuffield Department of Population Health, University of Oxford, Oxford, United Kingdom; Clinical Trial Service Unit & Epidemiological Studies Unit, Nuffield Department of Population Health, University of Oxford, Oxford, United Kingdom; Clinical Trial Service Unit & Epidemiological Studies Unit, Nuffield Department of Population Health, University of Oxford, Oxford, United Kingdom; Clinical Trial Service Unit & Epidemiological Studies Unit, Nuffield Department of Population Health, University of Oxford, Oxford, United Kingdom; Clinical Trial Service Unit & Epidemiological Studies Unit, Nuffield Department of Population Health, University of Oxford, Oxford, United Kingdom; Clinical Trial Service Unit & Epidemiological Studies Unit, Nuffield Department of Population Health, University of Oxford, Oxford, United Kingdom; Instituto Tecnológico y de Estudios Superiores de Monterrey, Monterrey, Mexico; Faculty of Medicine, National Autonomous University of Mexico, Mexico City, Mexico; Experimental Research Unit from the Faculty of Medicine, National Autonomous University of Mexico, Mexico City, Mexico; Clinical Trial Service Unit & Epidemiological Studies Unit, Nuffield Department of Population Health, University of Oxford, Oxford, United Kingdom; Faculty of Medicine, National Autonomous University of Mexico, Mexico City, Mexico; Clinical Trial Service Unit & Epidemiological Studies Unit, Nuffield Department of Population Health, University of Oxford, Oxford, United Kingdom

**Keywords:** adiposity, infection, Mexico, Mendelian randomization

## Abstract

**Background:**

The causal relevance of different adiposity markers to infection-related death remains uncertain. We estimated the lifelong associations of genetically predicted adiposity traits with infection-related mortality among 126 491 Mexican adults.

**Methods:**

Mendelian randomization (MR) using trans-ancestry instruments for body mass index (BMI), fat-mass percentage (FM%), and waist and hip circumference was used to estimate causal effects on infection-related mortality at ages 35–74 years, overall and across six infection subtypes. Multivariable MR was used to assess independent associations.

**Results:**

Genetic instruments explained 1.6%–3.5% of the variance in adiposity markers and the strengths of their associations with the markers were similar in men and women. Over 20 years, 2592 infection-related deaths occurred at ages 35–74 years. Genetically predicted adiposity was strongly associated with infection mortality, with the strongest association for FM% [hazard ratio (HR) per 1-SD higher level: 2.54; 95% confidence interval (CI) 1.91–3.38] and the weakest for hip circumference (HR 1.61; 95% CI 1.30–2.01). Associations were consistent across six subtypes: respiratory; urinary tract; gastrointestinal; sepsis; skin, bone, and connective tissue; and other infections. For BMI and FM%, mutual adjustment for waist and hip circumference reduced the associations by nearly one-third. For waist circumference, adjustment for BMI and hip circumference reduced the association by more than half, while, for hip circumference, mutual adjustment for BMI and waist circumference almost eliminated its association. Sensitivity analyses supported the main conclusions.

**Conclusion:**

Higher adiposity is causally associated with a higher risk of infection-related mortality in Mexican adults. Strategies to reduce the population levels of adiposity in Mexico could contribute to lowering mortality from infection.

Key Messages

- Obesity continues to rise globally and infections are a major cause of death, but the impact of different adiposity traits on infection remains uncertain.
- Using a Mendelian randomization approach applied to a large Mexican prospective cohort study, we found that 1-SD higher genetically predicted BMI, fat-mass percentage, and waist circumference were each associated with more than a doubling in infection-related mortality at ages 35–74 years, with similarly strong associations seen for all infection subtypes. The association of hip circumference with infection was less strong and was explained by its association with the other adiposity markers.
- These estimated causal effects of adiposity on infection reinforce the need to reduce population levels of adiposity in Mexico in order to prevent deaths from infection as well as other major causes of death.

## Introduction

Globally, >2 billion people are overweight or obese [i.e. they have a body mass index (BMI) of >25 kg/m^2^] and the prevalence of excess adiposity continues to rise [1]. Infections remain a major cause of mortality, accounting for an estimated 16 million deaths annually [2]. Excess adiposity may increase susceptibility to infection through several mechanisms [3], including by promoting type 2 diabetes, which is itself associated with a higher risk of infection and infection-related mortality [4, 5]. However, poorly controlled diabetes can also lead to weight loss, potentially distorting associations between adiposity and infection in conventional observational analyses [6]. Previous large observational studies have typically reported J- or U-shaped associations between adiposity and pneumonia and other respiratory infections, and log-linear associations with COVID-19, sepsis, and skin and urinary infections [7–10], but such studies remain susceptible to residual confounding, reverse causality, and other biases inherent in observational research.

Mendelian randomization (MR) approaches can help overcome these limitations through the use of genetic variants as instruments for adiposity, thereby strengthening causal inference [11]. However, existing MR studies have focused predominantly on BMI, a limited range of infection outcomes, and populations of European ancestry, and have yielded widely varying effect estimates [7, 12–14]. Clarifying the causal role of adiposity in infection is particularly important in populations with a high burden of obesity and diabetes. Mexico is one such setting, with among the highest prevalences of obesity, type 2 diabetes, and infection-related mortality worldwide [15, 16].

Using data from the Mexico City Prospective Study (MCPS) of 150 000 adults followed for two decades, we previously reported MR analyses showing that BMI was strongly associated with infection-related mortality and that diabetes appeared to mediate a substantial proportion of this association [17]. In the present study, we extend these analyses by investigating the associations of multiple genetically predicted adiposity markers with overall and cause-specific infection-related mortality.

## Methods

### Study design and participants

The design and methods of the MCPS have been described previously [18, 19]. Between 14 April 1998 and 28 September 2004, households in two urban districts (Coyoacán and Iztapalapa) were visited and residents aged ≥35 years were invited to participate. Details of recruitment, baseline assessments, construction of the genetic instruments, mortality follow-up, and statistical methods are provided in the Appendix; a summary is given below.

### Adiposity markers and mortality outcomes

Four adiposity markers were considered: BMI (general adiposity), fat-mass percentage (FM%; body-fat composition), waist circumference (abdominal adiposity), and hip circumference (gluteo-femoral adiposity). Infections were analysed overall and in six categories of underlying cause of death: sepsis without specified source; skin, bone, and connective tissue; gastrointestinal; urinary tract; respiratory; and other infections (Supplementary Table 1). Respiratory infections were further subdivided into pneumonia, coronavirus disease 2019 (COVID-19), and bronchitis including exacerbations of chronic obstructive pulmonary disease.

### Statistical analysis

Assuming an additive genetic model of inheritance, one-sample MR using the Wald ratio method [11] was used to assess the causal relevance of each adiposity marker to infection-related mortality. Sex-specific Cox models adjusted for age at risk (in 5-year groups) and the first seven genetic principal components (PCs) for ancestry were used to estimate the associations between each trans-ancestry adiposity genetic score (GS) [20] and mortality. Linear regression, adjusted for age, age-squared, and the first seven genetic PCs, was used to estimate the associations between each GS and its corresponding baseline adiposity marker. Wald ratio estimates were calculated separately for men and women, standardized to 1-SD higher adiposity, and combined by using inverse-variance weighting.

To assess whether the associations between genetically predicted adiposity and infectious-disease mortality were independent of other adiposity markers, multivariable Mendelian randomization (MVMR) analyses were conducted with each GS adjusted for the other adiposity GSs (correlations are shown in Supplementary Table 2). The GSs for BMI, waist circumference, and hip circumference were mutually adjusted, whereas the FM% GS was adjusted for the GSs for waist and hip circumference.

Cox models were also used to relate baseline adiposity (per 1-SD higher level) to the risk of death from all and specific infections. To reduce reverse causality—particularly weight loss due to poorly controlled diabetes—these analyses were restricted to participants without diabetes or other chronic diseases at baseline [6].

Sensitivity analyses included restriction to participants unrelated to the third degree; analyses stratified by age at risk, residential district, Indigenous American ancestry, physical activity, smoking, and alcohol consumption; alternative two-sample MR methods (inverse-variance-weighted, MR-PRESSO, weighted median, and MR–Egger) [11, 21]; and non-linear MR (doubly-ranked and residual methods) to assess departures from log-linearity [22, 23].

Analyses were conducted by using STATA (version 17.0) and R (version 4.4.2).

## Results

### Study population and baseline characteristics

Of the 159 755 participants recruited, 33 264 (21%) were excluded from the genetic analyses, leaving 126 491 participants for analysis. Exclusions included 12 116 (7.6%) participants aged ≥75 years at recruitment, a further 19 601 (12.3%) without quality-controlled acceptable genetic data, and a further 1547 (<1%) with uncertain mortality linkage (Supplementary Figure 1). For the observational analyses, 48 573 (30%) participants were excluded, leaving 111 182 participants for analysis. Exclusions included 12 116 (7.6%) aged ≥75 years at recruitment, a further 34 169 (21.4%) who had diabetes or another chronic diseases at recruitment, and a further 2288 (4.6%) with unreliable or missing data.

Among the 126 491 participants in the genetic-analysis population, the mean age was 51 years (11 SD), 68% were women, the mean Indigenous American ancestry percentage was 67% (18% SD), and 19% had previously diagnosed or undiagnosed diabetes (Table 1). The mean levels of BMI, FM%, waist circumference, and hip circumference among men were 28.0 kg/m^2^ (4.2 SD), 29% (4% SD), 96 cm (10 SD), and 101 cm (8 SD), respectively. The corresponding measures among women were 29.7 kg/m^2^ (5.2 SD), 43% (4% SD), 93 cm (12 SD), and 107 cm (11 SD), respectively. Men were more likely to have a higher level of education and to report leisure-time physical activity than women, but were also more likely to smoke tobacco and drink alcohol. The characteristics of the participants without diabetes or other chronic diseases who contributed to the observational-analysis population are shown in Supplementary Table 3.

**Table 1:** Baseline characteristics of 126 491 participants aged 35–74 years at recruitment (genetic-analysis population).

	Men	Women	Overall
No. of participants	40 829	85 662	126 491
Age, ancestry, and socio-economic factors			
Age (years)	51 (11)	50 (11)	51 (11)
Indigenous American ancestry (%)	67 (18)	67 (18)	67 (18)
Resident of Coyoacán	16 987 (42)	31 495 (37)	48 482 (38)
University or high-school educated	10 012 (25)	10 069 (12)	20 081 (16)
Lifestyle factors			
Current smoker	20 797 (51)	20 323 (24)	41 120 (33)
Current alcohol drinker	31 639 (78)	53 740 (63)	85 379 (68)
Physical activity at least one time/week	12 321 (30)	15 976 (19)	28 297 (22)
Anthropometry, blood pressure, and HbA1c			
Height (cm)	165 (7)	152 (6)	156 (6.5)
Weight (kg)	76 (13)	68 (13)	72 (13)
BMI (kg/m^2^)	28.0 (4.2)	29.7 (5.2)	28.8 (4.7)
Fat mass (%)	29 (4.4)	43 (4.0)	36 (4.2)
Waist circumference (cm)	96 (10)	93 (12)	94 (11)
Hip circumference (cm)	101 (8)	107 (11)	104 (9)
Blood pressure (mmHg)			
Systolic	128 (15)	126 (17)	127 (16)
Diastolic	84 (10)	82 (10)	83 (10)
HbA1c (%)	6.1 (1.7)	6.1 (1.7)	6.1 (1.7)
Medical history			
Diabetes^a^	7707 (19)	15 743 (18)	23 450 (19)
Ischaemic heart disease	733 (1.80)	895 (1.04)	1628 (1.29)
Stroke	374 (0.9)	789 (0.9)	1163 (0.9)
Other^b^	2025 (5.0)	8215 (9.6)	10 240 (8.1)

Mean (SD) or *n* (%). HbA1c, glycosylated haemoglobin A1c.

a Previously diagnosed self-reported diabetes or measured HbA1c of ≥6.5%.

b Chronic kidney disease, cirrhosis, cancer, or emphysema.

### Genetic instruments for adiposity

**Figure 1:**
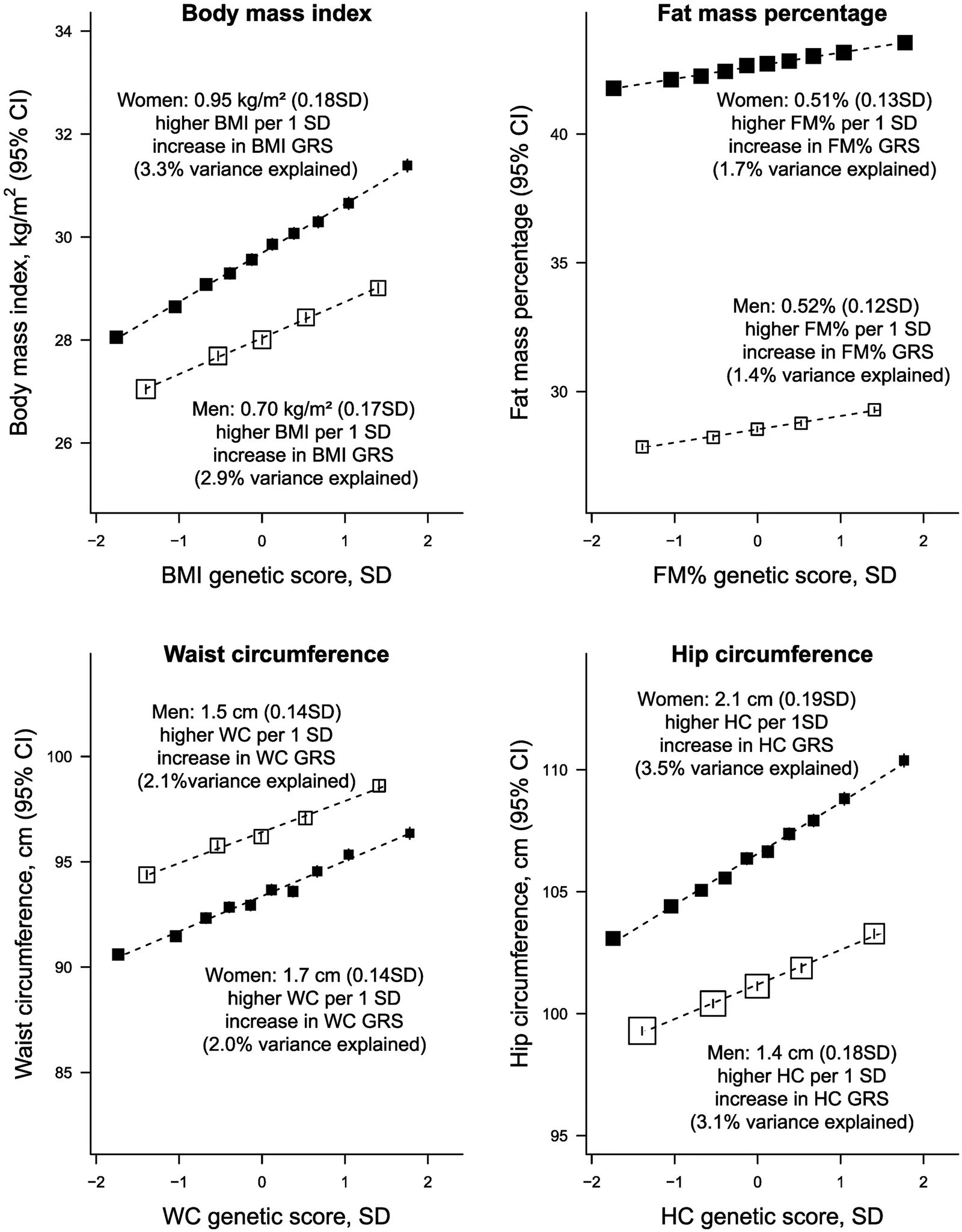
Mean adiposity levels by GS in men and women aged 35–74 years at recruitment. Mean baseline adiposity levels across five equally sized groups of the corresponding GSs in men (white squares) and 10 equally sized groups in women (black squares). The F-statistics for the GSs in men were 1220 for BMI, 565 for FM%, 854 for WC, and 1325 for HC. The corresponding F-statistics in women were 2962, 1464, 1705, and 3099, respectively. CI, confidence interval; HC, hip circumference; WC, waist circumference.

The genetic instruments from the trans-ancestry meta-analyses comprised 724 single-nucleotide polymorphisms (SNPs) for BMI, 636 SNPs for FM%, 521 SNPs for waist circumference, and 651 SNPs for hip circumference. The per-allele effects of these SNPs on the adiposity markers in MCPS correlated moderately well with the estimates from the trans-ancestry meta-analyses, with the weakest correlation observed for FM% in men (Supplementary Figure 2). The adiposity instruments explained between 1.4% and 3.5% of the variance in the corresponding measured adiposity marker (Fig. 1), with F-statistics ranging from 565 to 3100, and were largely unrelated to a range of baseline characteristics (Supplementary Tables 4–7). A notable exception was diabetes—a strong mediator of BMI and risk of death in this population [17]—that was strongly predicted by both the BMI and FM% instruments. For each of the four genetic instruments, the association between it and the adiposity marker that it was designed to predict was continuous and linear, with broadly similar strengths of association in men and women (Fig. 1).

### Genetically predicted adiposity and infection-related mortality

During a median of 20 years of follow-up, 2592 of the 126 491 participants in the genetic-analysis population died from infection at ages 35–74 years, including 1641 deaths from respiratory infection, 227 from urinary tract infection, 226 from gastrointestinal infection, 197 from sepsis, 126 from skin, bone, or connective tissue infection, and 175 from other infections. In the observational-analysis population, there were 1942 deaths from infection with a similar proportional distribution by type.

**Figure 2:**
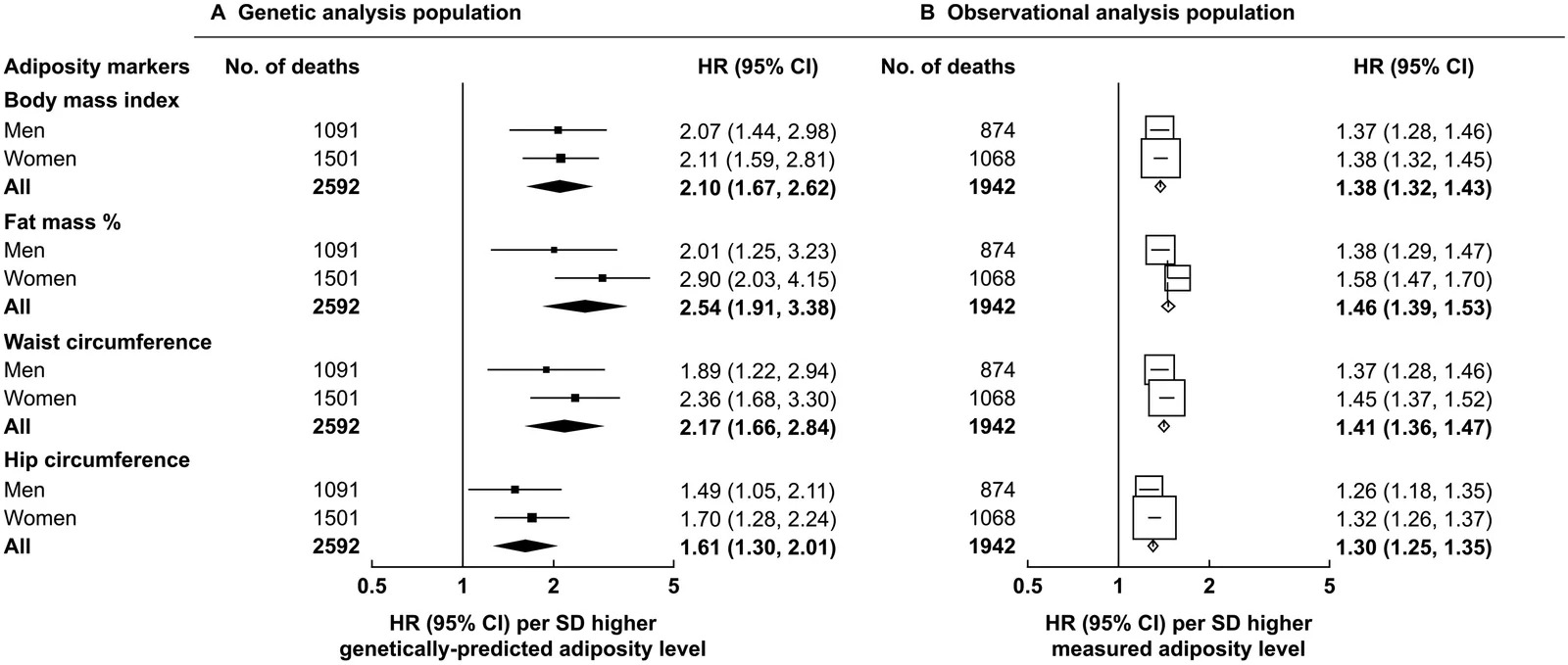
Associations of genetically predicted and observed adiposity markers with infection-related death at ages 35–74 years. Estimates show hazard ratios (HRs) and 95% confidence intervals (CIs). MR analyses use the ratio method and are adjusted for age at risk and seven PCs of genetic ancestry. Observational analyses were restricted to participants without diabetes or other chronic diseases at baseline, and were additionally adjusted for residential district, education, physical exercise, and smoking and drinking status. Adiposity markers were modelled per 1-SD increase across the full, sex-specific distributions.

Figure 2 presents the overall and sex-specific hazard ratios (HRs) for infection-related death per 1-SD higher genetically predicted adiposity level and, for the subset of participants without diabetes or other chronic diseases, observational analyses relating adiposity to infection risk. In the genetic-analysis population, all four adiposity markers were strongly associated with the risk of death from infection, with the strongest association observed for FM% [HR per 1-SD higher genetically predicted level: 2.54, 95% confidence interval (CI) 1.91–3.38], followed by waist circumference (HR 2.17, 95% CI 1.66–2.84), BMI (HR 2.10, 95% CI 1.67–2.62), and hip circumference (HR 1.61, 95% CI 1.30–2.01). In each case, associations were similar in men and women. These associations were substantially larger than those observed for measured baseline levels of these adiposity markers in the observational-analysis population, which was restricted to those without diabetes or other chronic diseases at recruitment (Supplementary Figures 3 and 4).

### Impact of mutual adjustment for other adiposity markers

**Figure 3:**
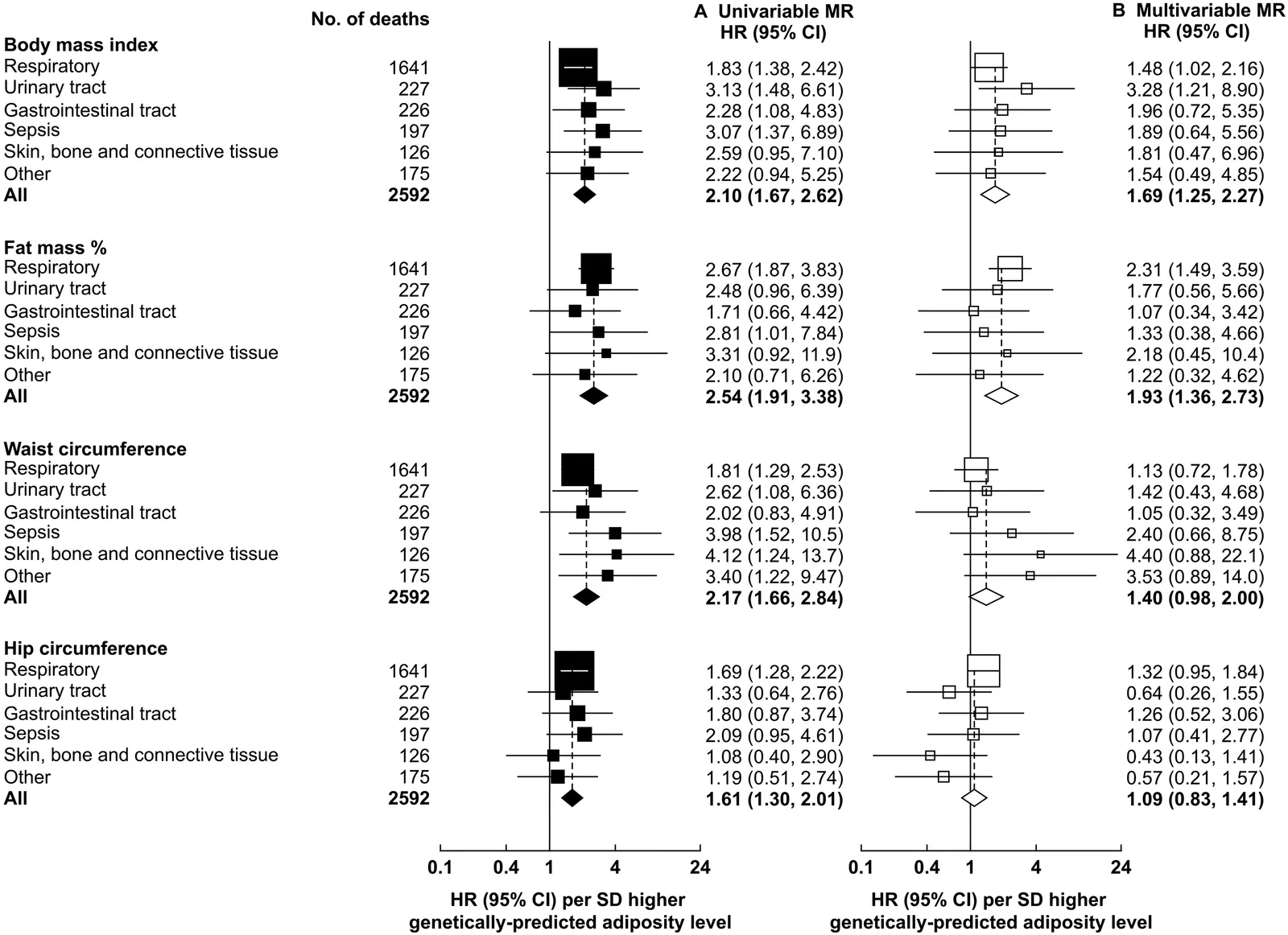
Associations of genetically predicted adiposity markers with cause-specific infection-related death at ages 35–74 years. (a) Analyses conducted as in the genetic analyses of Fig. 2, but stratified by infection type. (b) Analyses additionally adjusted for other adiposity markers: BMI and FM% are adjusted for waist and hip circumference, while waist and hip circumference are adjusted for each other and for BMI.

In the genetic-analysis population, associations between genetically predicted adiposity markers and infection-related death were attenuated after mutual adjustment for the other adiposity GSs (Fig. 3). Attenuation was greatest for hip circumference, where adjustment for the BMI and waist circumference instruments reduced the log hazard ratio by more than four-fifths, and smallest for BMI and FM%, where adjustment for the waist and hip circumference instruments reduced the log HRs by less than one-third. Consequently, in the MVMR analyses, FM% remained the adiposity marker most strongly associated with infection-related death (HR per 1-SD higher genetically predicted level: 1.93, 95% CI 1.36–2.73).

### Specific infection subcategories

The joint and independent associations of genetically predicted adiposity markers with cause-specific infection-related death are shown in Fig. 3. For FM% (the adiposity marker most strongly associated with overall infection-related mortality), associations were broadly similar across infection subtypes, although the CIs were wide. Similar patterns were observed when respiratory infections were subdivided into pneumonia, COVID-19, and bronchitis (Supplementary Figure 5). Waist circumference showed weaker associations with overall infection mortality than FM%, largely due to a weaker association with respiratory infections (Fig. 3). Infection-specific results by sex are provided in Supplementary Figure 6 and were broadly similar in both sexes.

### Sensitivity analyses

Associations of genetically predicted adiposity with the risk of death from infection were broadly similar when analyses were repeated in the subset of participants unrelated to the third degree (Supplementary Figure 7). For FM%, the association with the risk of death from infection was weaker at older ages and among individuals with the highest proportion of Indigenous American ancestry, but was similar across residential districts and across lifestyle risk factors, including smoking, alcohol-consumption, and physical-activity categories (Supplementary Figure 8, which also presents subgroup-specific results for the other adiposity markers). Estimates from two-sample MR analyses (MR-PRESSO, weighted median, and MR–Egger) were directionally consistent with those of the main analysis, but more modest in magnitude, particularly for FM%, waist circumference, and hip circumference among men (Supplementary Table 8). Analyses in which the residual and doubly-ranked non-linear methods were used supported the inherently assumed log-linear relationships (Supplementary Figure 9).

## Discussion

In this MR study of ∼126 500 Mexican adults, higher genetically predicted adiposity was associated with an increased risk of infectious-disease mortality before age 75 years. Associations were strongest for FM% and weakest for hip circumference, and were largely consistent across infectious-disease subtypes and population subgroups. In mutually adjusted analyses, the association with FM% was least attenuated, and it remained independently and strongly associated with infectious-disease mortality, whereas the association with hip circumference appeared to be explained by its correlation with other adiposity markers.

Our findings are consistent with those of previous MR studies in predominantly European populations linking lifelong excess adiposity with infectious-disease outcomes. In UK Biobank, each 4.8-kg/m^2^ higher genetically predicted BMI was associated with modestly higher risks of infectious-disease hospitalization, including pneumonia, sepsis, urinary tract infections, and skin and soft-tissue infections [12]. In the Norwegian HUNT, compared with a genetically predicted BMI of 25 kg/m^2^, a genetically predicted BMI of 30 kg/m^2^ was associated with an HR of 1.78 (95% CI 1.40–2.27) for bloodstream infection (BSI) incidence and 2.56 (95% CI 1.31–4.99) for BSI mortality [13]. Analyses combining UK Biobank and the HUNT study also found that a 1-SD higher genetically predicted BMI value was associated with a 47% increase in the odds of hospitalization for COVID-19 [24], while, in the Copenhagen General Population Study, each 5-kg/m^2^ higher genetically predicted BMI was associated with a 28% increase in the odds of any infection, particularly urinary tract and skin infections, although not pneumonia or sepsis [7].

Compared with these studies, the associations between genetically predicted adiposity and infectious-disease mortality were substantially stronger in this Mexican population. Some of this difference may reflect our focus on mortality rather than a combination of fatal and non-fatal infectious-disease events. However, differences in population characteristics may have also contributed. Mexico has a substantially higher prevalence of obesity and type 2 diabetes than most European populations and diabetes may amplify the adverse consequences of excess adiposity on infection risk. For example, in our study, each 1-SD higher genetically predicted BMI value (∼4.7 kg/m^2^) was associated with more than a doubling in the risk of infectious-disease mortality and more than a trebling in the risk of sepsis and urinary tract infections. Although these associations were attenuated by nearly one-third after adjustment for genetically predicted body-fat distribution, BMI remained strongly associated with infection-related mortality. Associations for FM% were even stronger and were less affected by adjustment for the other adiposity markers. Even after mutual adjustment, each 1-SD higher genetically predicted FM% (∼4.2%) was associated with nearly twice the risk of infectious-disease mortality, including more than a doubling of the risk of respiratory infections.

The estimates from our MR analyses were substantially stronger than those from the conventional epidemiological analyses relating adiposity markers to infection-related mortality among participants without diabetes or other chronic diseases at recruitment. This difference is expected because, to minimize reverse causality in the observational analyses—particularly the impact of poorly controlled diabetes on weight loss [6]—we excluded those with diabetes, but, in so doing, removed an important pathway through which adiposity may increase infection-related mortality. As previously reported, analyses in this cohort suggest that diabetes may mediate a substantial proportion of the excess risk of infectious-disease death attributable to adiposity [17]. In addition, the observational analyses did not correct for regression dilution bias [25]; although this is unlikely to have materially affected BMI, given its high within-person repeatability, it may have led to greater underestimation for other adiposity markers. Finally, the stronger associations in the MR analyses may also reflect their estimation of the lifelong effects of adiposity on infection risk [26].

Taken together, our findings, alongside previous MR evidence, provide strong support for a causal association between lifelong excess adiposity and infection-related mortality. The consistency of these findings across populations strengthens the evidence for causality, while the larger effect sizes observed in this Mexican cohort suggest that the adverse consequences of adiposity may be greatest in populations with a high burden of obesity and type 2 diabetes.

Several mechanisms may underlie the observed associations between adiposity and infectious-disease mortality. Previous work in this cohort has suggested that diabetes is an important mediator of the association between adiposity and mortality risk [17], while MR analyses have also reported positive associations between genetic liability to type 2 diabetes and risk of death from infection [27]. Excess adiposity may further increase susceptibility to infection through metabolic dysregulation, chronic low-grade inflammation, and impaired immune function [3, 28]. Adiposity-related reductions in lung function and ventilatory reserve, together with the potential role of adipose tissue as a reservoir for ACE2-binding pathogens such as SARS-CoV-2, may contribute to respiratory infections [28]. Excess adiposity has also been linked to structural and functional changes affecting the skin, lymphatic, and vascular systems that may increase susceptibility to sepsis [3], while alterations in gut permeability and the intestinal microbiome may contribute to gastrointestinal infections [29].

Our findings have implications for public health and clinical practice. Although current guidelines in Mexico already emphasize weight reduction primarily for the prevention of cardiovascular disease [30], our results suggest that reducing excess adiposity could also contribute to lowering the burden of severe infectious disease. Individuals with obesity may therefore warrant prioritization for preventive measures, including vaccination during infectious-disease outbreaks. These findings are likely to be relevant beyond Mexico, particularly in populations with high prevalences of obesity and diabetes, including Mexican American populations in the USA.

The chief strengths of this study include its large sample size, prolonged follow-up, and focus on a previously understudied ancestry group with high prevalences of obesity and diabetes. To our knowledge, it is the first MR study in a Latin American population to have examined the causal relevance of multiple genetically predicted adiposity markers across a wide range of infectious causes of death. Genetic data were available for nearly all participants, allowing a one-sample MR approach, and the use of allele scores as the primary instruments helped minimize weak-instrument bias. The instruments were derived from trans-ancestry genome-wide association studies (GWAS) that did not include MCPS and, although those GWAS included relatively few Hispanic participants, the instruments were strongly associated with the corresponding adiposity markers in this cohort, despite not all constituent variants being individually nominally associated with these markers.

Several limitations should be acknowledged. Although the sensitivity analyses yielded consistent results, some residual horizontal pleiotropy cannot be excluded. The use of predicted fat mass (rather than a direct measure of fat mass) may have resulted in underestimation of the true instrument-to-fat-mass association and, therefore, overestimation of the association between fat mass and infection-related mortality. Despite the large overall sample size, statistical power was limited for some cause-specific infectious-disease outcomes, resulting in imprecise estimates. The sensitivity non-linear MR analyses should also be interpreted cautiously given the recent concern over the use of such methods [31]. Indeed, previous non-linear MR analyses conducted in the same cohort revealed non-zero associations between genetically predicted BMI and both age and sex [17]. The restriction to participants aged ≥35 years precludes the assessment of infections occurring earlier in life. Cause of death was ascertained from death certificates and some misclassification of specific infectious causes was therefore inevitable. The study population was recruited from just two districts of Mexico City and is not representative of adults throughout Mexico. However, prospective studies of non-representative cohorts of individuals can provide reliable and widely generalizable evidence on associations between risk factors and disease [32, 33]. Finally, the lack of information on non-fatal infectious diseases means that our conclusions apply directly only to mortality.

## Conclusions

In summary, our findings provide strong evidence that excess adiposity is a causal risk factor for death from infection in Mexican adults. The findings underscore the need for integrated strategies addressing the risks of both communicable and non-communicable diseases due to adiposity in Mexico and elsewhere.

## Ethics approval

Ethics approval was obtained from the Mexican Ministry of Health, the Mexican National Council for Science and Technology (reference 0595P-M), and the University of Oxford (reference C99.260). All participants provided written informed consent.

## Supplementary Material

dyag197_Supplementary_Data

## Data Availability

Data from the MCPS are available to bona fide academic researchers. For more details, the study’s Data and Sample Sharing policy may be viewed (in English or Spanish) at https://www.ctsu.ox.ac.uk/research/mcps. Available study data can be examined in detail through the study’s Data Showcase, available at https://datashare.ndph.ox.ac.uk/mexico/. MCPS ancestry-specific allele frequencies are available in a public browser (https://rgc-mcps.regeneron.com/). For the purpose of open access, the author has applied a CC BY public copyright licence to any Author Accepted Manuscript version arising from this submission.
