# Supplementary material for: Adiposity and infection-related mortality: Mendelian randomization study of 126 000 Mexican adults
Source: Int J Epidemiol. 2026 Sep 11;55(5):dyag197. doi: 10.1093/ije/dyag197 (PMC13568476; doi:10.1093/ije/dyag197)
Supplement: Supplementary file 1 [file dyag197_supplementary_data.zip › ije-2026-03-0508-File006.pdf]

Adiposity and infection-related mortality:
Mendelian randomisation study of 126,000 Mexican adults

Online appendix

Page

Webfigures

1. Participant flow diagram 2
2. Comparison of the effect sizes of variants associated with adiposity measures in the trans-ancestry meta-analysis and MCPS 3

Associations of measured adiposity markers with:

3. Infection-related mortality at ages 35-74 years, across fifths of the adiposity distributions 4
4. Cause-specific infection-related mortality at ages 35-74 years 5

Associations of genetically-predicted adiposity markers with:

5. Subtypes of respiratory infectious disease mortality at ages 35-74 years 6
6. Cause-specific infection-related mortality at ages 35-74 years, by sex 7
7. Infection-related mortality at ages 35-74 years in the subset of unrelated participants 8
8. Infection-related mortality at ages 35-74 years, by selected baseline characteristics 9
9. Infection-related mortality across strata of adiposity markers using the residual and doubly-ranked method 10

Webtables

1. ICD-10 classification of infectious disease deaths at ages 35-74 years (genetic analysis population), by underlying cause 11
2. Sex-specific correlations between: a) the genetic instruments for adiposity; and b) the measured levels of adiposity 12
3. Baseline characteristics of 111,182 participants (observational analysis population) aged 35-74 years at recruitment, by sex 13

Baseline characteristics of 126,491 participants (genetic analysis population) aged 35-74 years at recruitment, by fifth of genetic score for:

4. BMI 14
5. Fat mass percentage 15
6. Waist circumference 16
7. Hip circumference 17

8. Sensitivity ‘two-sample’ MR analyses for death from infection at ages 35-74 years, using SNP-to-adiposity associations derived from a trans-ancestry GWAS of China Kadoorie Biobank and UK Biobank 18

Supplementary methods and references 19

Figure S1: Participant flow diagram

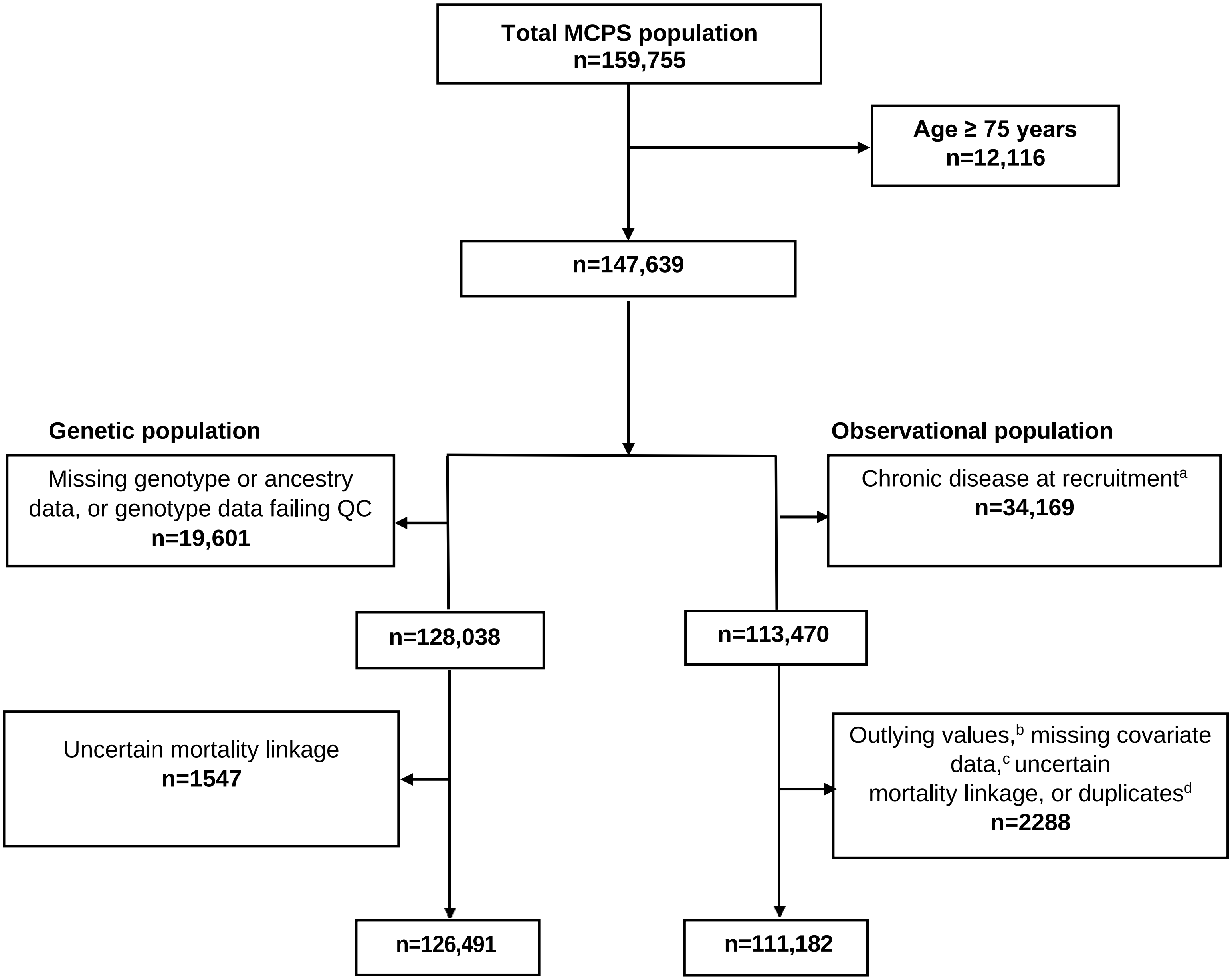

[a] Diabetes, ischaemic heart disease, stroke, cancer, chronic kidney disease, cirrhosis, or emphysema. [b] Outlying values of anthropometry were pre-defined as: height <120 or >200 cm, weight <35 or >250 kg, waist circumference <60 or >180 cm, hip circumference <70 or >180 cm, waist-to-hip ratio <0.5 or >1.5, or BMI <15 or >60 kg/m^2. [c] Age, sex, district of residence, education, alcohol drinking, physical activity, and smoking. [d] Participants who were recruited more than once. BMI = body mass index. QC= quality control. MCPS= Mexico City Prospective Study.

Figure S2. Comparison of the effect sizes of variants associated with adiposity measures in the trans-ancestry meta-analysis and Mexico City Prospective Study

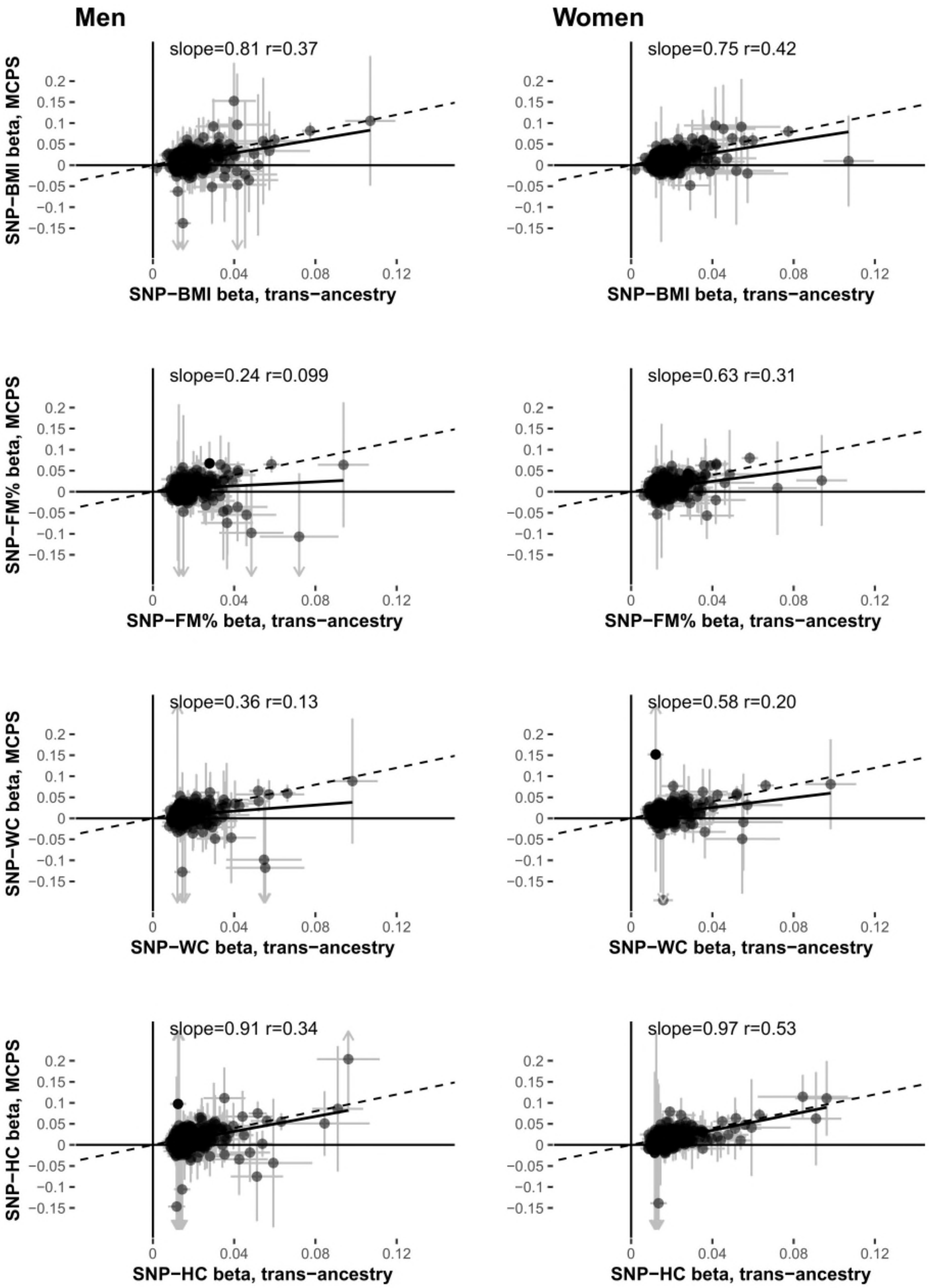

Adiposity measures were transformed by applying rank inverse normalisation to the residuals from a regression of adiposity on age and sex. The beta coefficients shown are SD units of adiposity in men or women respectively, and the vertical and horizontal grey lines through them represent 95% confidence intervals. The black sloped line shows the line of best fit from an inverse-variance-weighted linear regression. The dashed black line indicates the line y=x. r is the Pearson correlation coefficient. SD=standard deviation; SNP=single nucleotide polymorphism.

Figure S3. Associations of measured adiposity markers with infection related mortality at ages 35−74 years across fifths of their distributions

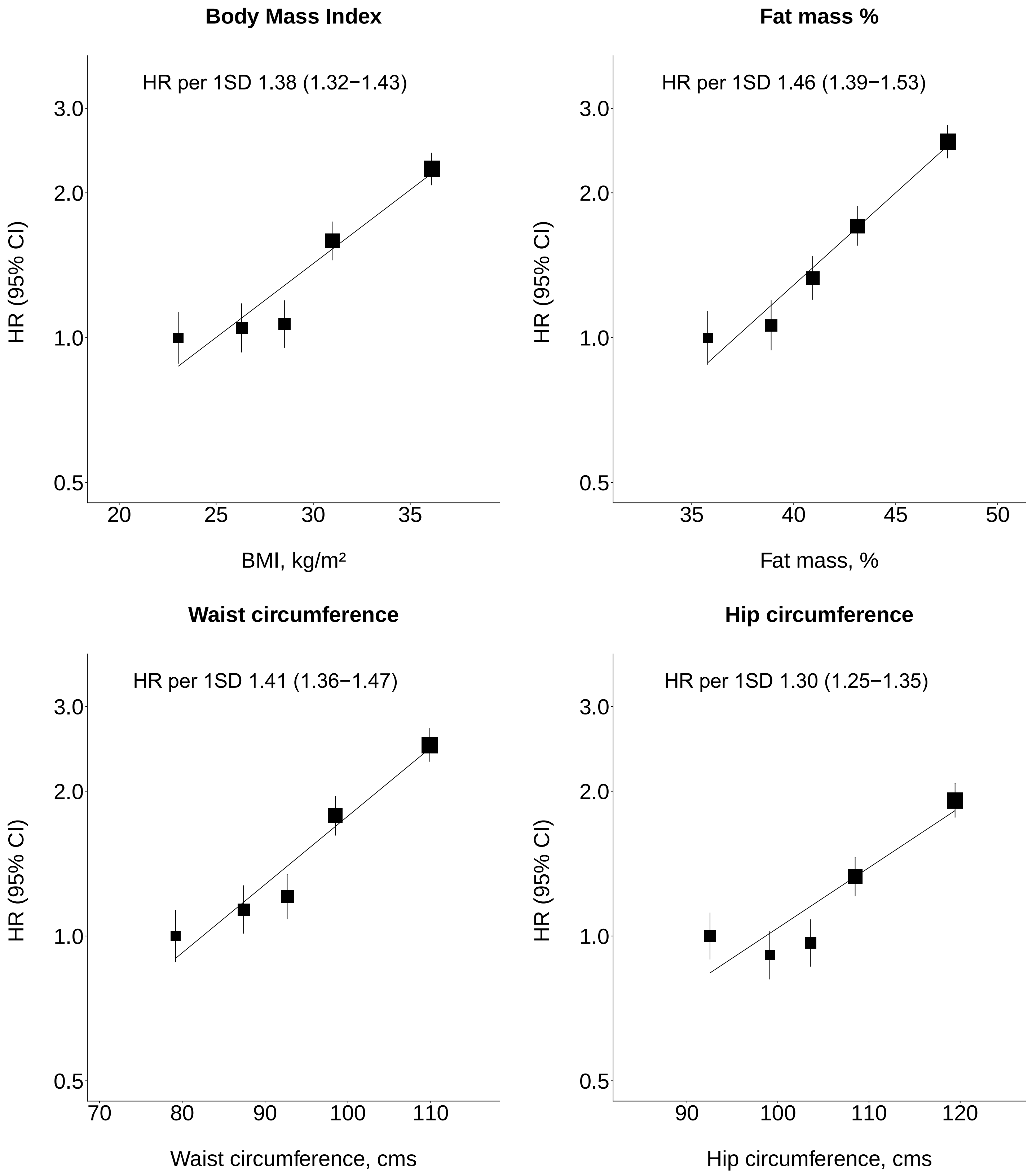

Estimates show hazard ratios (HR) and 95% confidence intervals (CI) across observed adiposity markers grouped as the five equally−sized groups of the sex−specific distributions. Analyses were adjusted for age−at−risk, sex, district, smoking, drinking, education and physical activity, and excluded those with diabetes or other chronic disease at recruitment.

Figure S4: Association of measured adiposity markers with cause-specific infection related mortality at ages 35-74 years

	No. of deaths	Overall HR (95% CI)
**Body mass index**		
COVID-19	783	1.55 (1.46, 1.64)
Pneumonia	483	1.25 (1.16, 1.36)
Bronchitis*	89	1.30 (1.08, 1.58)
**Respiratory**	**1355**	**1.42 (1.36, 1.49)**
Urinary tract	133	1.42 (1.23, 1.63)
Gastrointestinal tract	155	1.34 (1.17, 1.53)
Sepsis	96	1.32 (1.11, 1.58)
Skin, bone, connective tissue	70	1.58 (1.31, 1.91)
Other	133	0.95 (0.80, 1.13)
**All**	**1942**	**1.38 (1.32, 1.43)**
**Fat mass %**		
COVID-19	783	1.68 (1.55, 1.81)
Pneumonia	483	1.26 (1.14, 1.39)
Bronchitis*	89	1.38 (1.12, 1.72)
**Respiratory**	**1355**	**1.49 (1.41, 1.58)**
Urinary tract	133	1.52 (1.26, 1.84)
Gastrointestinal tract	155	1.55 (1.30, 1.83)
Sepsis	96	1.34 (1.09, 1.64)
Skin, bone, connective tissue	70	1.84 (1.47, 2.30)
Other	133	1.00 (0.83, 1.20)
**All**	**1942**	**1.46 (1.39, 1.53)**
**Waist circumference**		
COVID-19	783	1.51 (1.42, 1.61)
Pneumonia	483	1.29 (1.18, 1.40)
Bronchitis*	89	1.41 (1.17, 1.71)
**Respiratory**	**1355**	**1.42 (1.35, 1.49)**
Urinary tract	133	1.48 (1.27, 1.71)
Gastrointestinal tract	155	1.47 (1.28, 1.69)
Sepsis	96	1.43 (1.20, 1.70)
Skin, bone, connective tissue	70	1.77 (1.48, 2.11)
Other	133	1.07 (0.90, 1.27)
**All**	**1942**	**1.41 (1.36, 1.47)**
**Hip circumference**		
COVID-19	783	1.42 (1.35, 1.50)
Pneumonia	483	1.21 (1.11, 1.30)
Bronchitis*	89	1.28 (1.07, 1.52)
**Respiratory**	**1355**	**1.33 (1.27, 1.39)**
Urinary tract	133	1.36 (1.19, 1.55)
Gastrointestinal tract	155	1.25 (1.10, 1.43)
Sepsis	96	1.23 (1.05, 1.45)
Skin, bone, connective tissue	70	1.50 (1.27, 1.76)
Other	133	0.98 (0.83, 1.16)
**All**	**1942**	**1.30 (1.25, 1.35)**

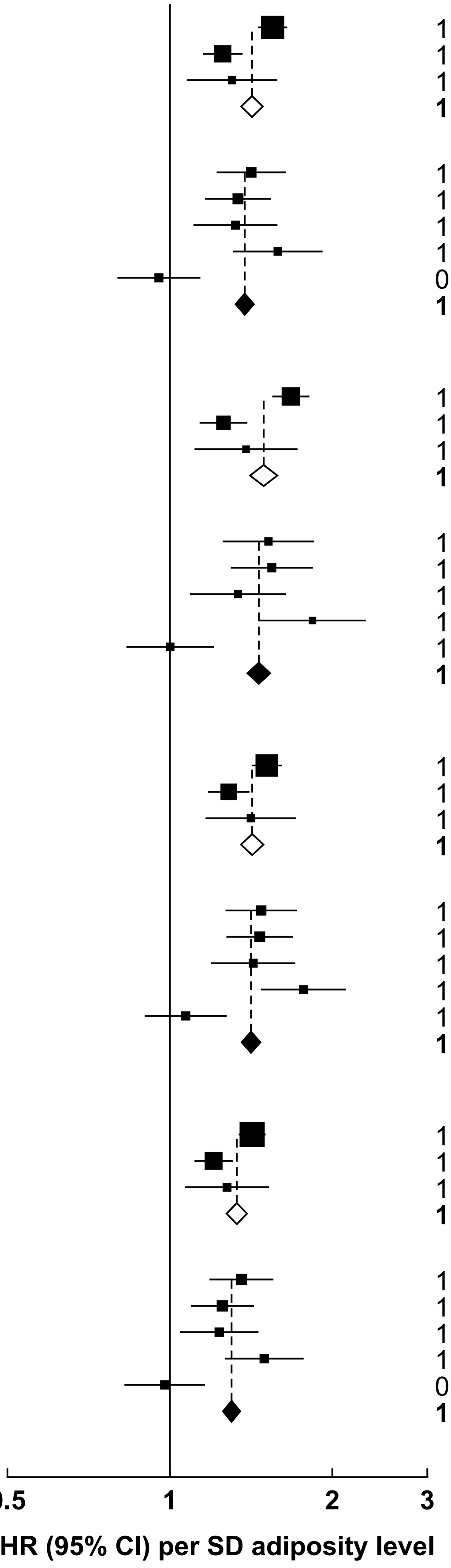

Observational analyses as for right hand side of Figure 2, but for specific infection categories.
* Includes COPD exacerbations.

Figure S5. Associations of genetically-predicted adiposity markers with subtypes of respiratory infectious disease mortality at ages 35-74 years

	No. of deaths	Univariable MR HR (95% CI)	Multivariable MR HR (95% CI)
**Body mass index**			
COVID-19	799	1.77 (1.18, 2.66)	1.47 (0.85, 2.52)
Pneumonia	711	2.07 (1.35, 3.17)	1.75 (0.99, 3.10)
Bronchitis*	131	1.21 (0.45, 3.22)	0.73 (0.20, 2.70)
**Any respiratory**	**1641**	**1.83 (1.38, 2.42)**	**1.48 (1.02, 2.16)**
**Fat mass %**			
COVID-19	799	2.78 (1.65, 4.68)	2.67 (1.20, 5.94)
Pneumonia	711	2.46 (1.43, 4.22)	1.93 (1.00, 3.74)
Bronchitis*	131	3.46 (0.99, 12.0)	3.40 (0.74, 15.6)
**Any respiratory**	**1641**	**2.67 (1.87, 3.83)**	**2.31 (1.49, 3.59)**
**Waist circumference**			
COVID-19	799	1.75 (1.08, 2.85)	1.14 (0.59, 2.19)
Pneumonia	711	1.97 (1.19, 3.28)	1.17 (0.59, 2.31)
Bronchitis*	131	1.39 (0.43, 4.48)	0.86 (0.18, 4.17)
**Any respiratory**	**1641**	**1.81 (1.29, 2.53)**	**1.13 (0.72, 1.78)**
**Hip circumference**			
COVID-19	799	1.61 (1.08, 2.40)	1.27 (0.78, 2.05)
Pneumonia	711	1.65 (1.09, 2.50)	1.17 (0.71, 1.94)
Bronchitis*	131	2.55 (0.98, 6.65)	3.23 (1.01, 10.3)
**Any respiratory**	**1641**	**1.69 (1.28, 2.22)**	**1.32 (0.95, 1.84)**

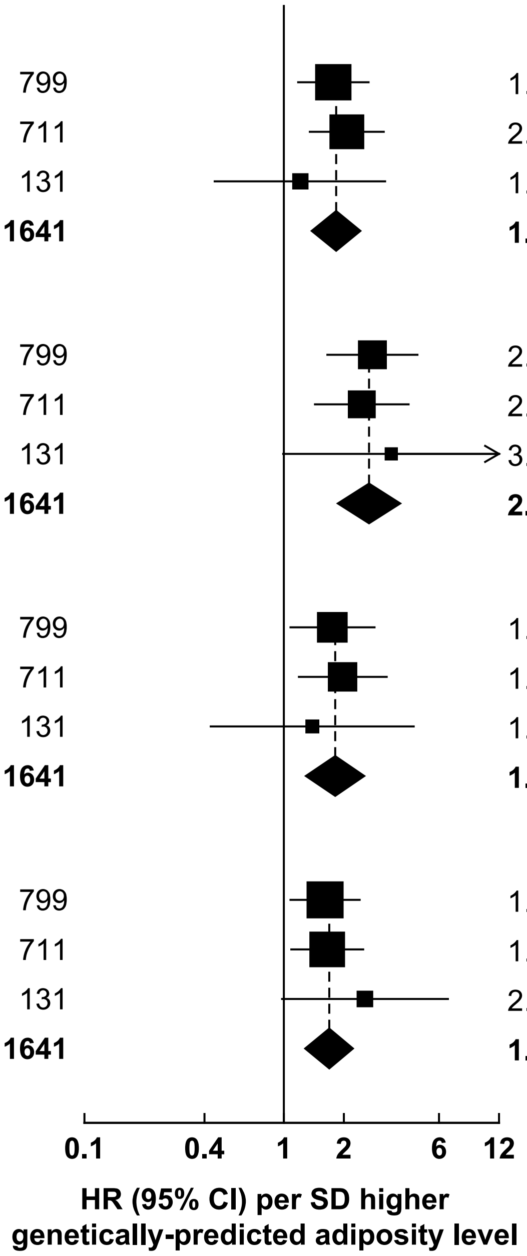

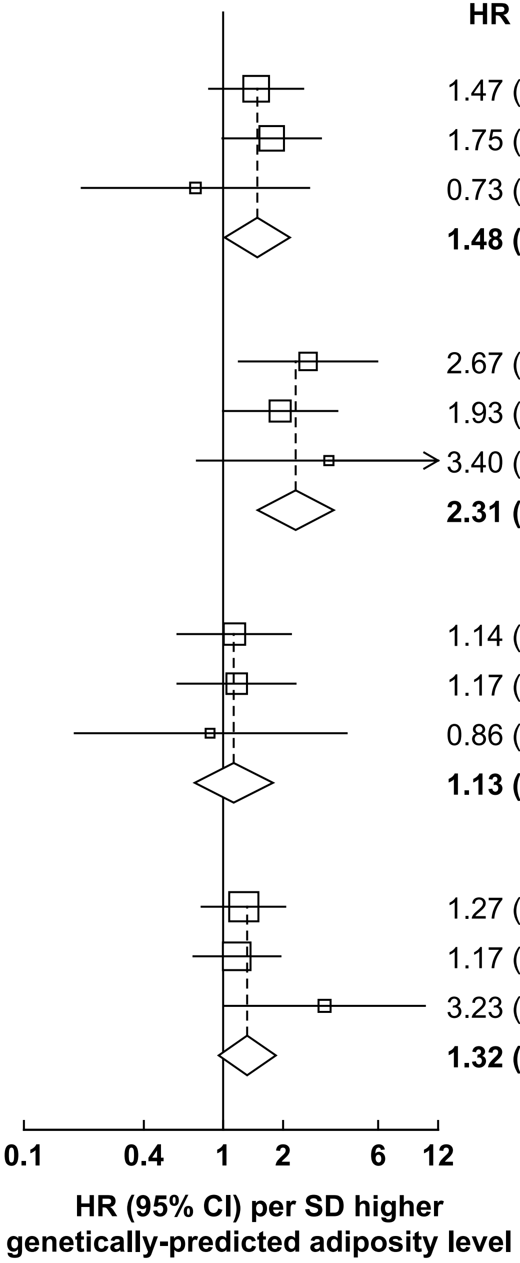

Mendelian randomisation analyses as per Figure 3. *Includes COPD exacerbations. COPD=Chronic Obstructive Pulmonary Disease.

Figure S6. Associations of genetically-predicted adiposity markers with cause-specific infection-related mortality at ages 35-74 years, by sex

	No. deaths in men	MR HR (95% CI) in men	No. deaths in women	MR HR (95% CI) in women
**Body mass index**				
COVID-19	385	1.71 (0.92, 3.17)	414	1.81 (1.05, 3.12)
Pneumonia	291	2.18 (1.08, 4.40)	420	2.01 (1.18, 3.44)
Urinary tract	76	2.17 (0.56, 8.42)	151	3.68 (1.50, 8.99)
Gastrointestinal tract	75	4.40 (1.11, 17.4)	151	1.73 (0.70, 4.22)
Sepsis	78	2.80 (0.72, 10.9)	119	3.23 (1.18, 8.84)
Bronchitis*	56	0.96 (0.20, 4.67)	75	1.39 (0.40, 4.88)
Skin, bone and connective tissue	53	2.27 (0.44, 11.7)	73	2.81 (0.79, 10.1)
Other	77	2.63 (0.68, 10.2)	98	1.99 (0.66, 6.02)
**All**	**1091**	**2.07 (1.44, 2.98)**	**1501**	**2.11 (1.59, 2.81)**
**Fat mass %**				
COVID-19	385	2.55 (1.14, 5.72)	414	2.96 (1.50, 5.84)
Pneumonia	291	2.48 (0.99, 6.21)	420	2.45 (1.26, 4.78)
Urinary tract	76	0.64 (0.11, 3.78)	151	4.25 (1.39, 13.0)
Gastrointestinal tract	75	1.44 (0.24, 8.59)	151	1.83 (0.60, 5.61)
Sepsis	78	3.37 (0.57, 19.8)	119	2.56 (0.73, 9.02)
Bronchitis*	56	1.68 (0.21, 13.3)	75	5.22 (1.09, 24.9)
Skin, bone and connective tissue	53	1.70 (0.20, 14.4)	73	4.79 (0.97, 23.6)
Other	77	1.06 (0.18, 6.20)	98	3.21 (0.80, 12.8)
**All**	**1091**	**2.01 (1.25, 3.23)**	**1501**	**2.90 (2.03, 4.15)**
**Waist circumference**				
COVID-19	385	1.75 (0.83, 3.69)	414	1.75 (0.92, 3.33)
Pneumonia	291	1.69 (0.72, 3.97)	420	2.15 (1.14, 4.03)
Urinary tract	76	1.92 (0.37, 9.99)	151	2.97 (1.03, 8.52)
Gastrointestinal tract	75	2.22 (0.42, 11.7)	151	1.94 (0.68, 5.57)
Sepsis	78	3.56 (0.68, 18.6)	119	4.22 (1.29, 13.8)
Bronchitis*	56	0.60 (0.09, 4.13)	75	2.27 (0.52, 9.84)
Skin, bone and connective tissue	53	4.83 (0.66, 35.3)	73	3.76 (0.84, 16.9)
Other	77	2.46 (0.47, 12.7)	98	4.18 (1.13, 15.5)
**All**	**1091**	**1.89 (1.22, 2.94)**	**1501**	**2.36 (1.68, 3.30)**
**Hip circumference**				
COVID-19	385	2.02 (1.12, 3.67)	414	1.34 (0.79, 2.29)
Pneumonia	291	1.10 (0.56, 2.15)	420	2.11 (1.25, 3.58)
Urinary tract	76	0.87 (0.24, 3.21)	151	1.61 (0.67, 3.89)
Gastrointestinal tract	75	1.52 (0.41, 5.67)	151	1.94 (0.80, 4.68)
Sepsis	78	3.00 (0.81, 11.1)	119	1.70 (0.63, 4.59)
Bronchitis*	56	3.44 (0.75, 15.7)	75	2.10 (0.61, 7.20)
Skin, bone and connective tissue	53	0.43 (0.09, 2.10)	73	1.93 (0.55, 6.79)
Other	77	1.18 (0.32, 4.34)	98	1.19 (0.40, 3.56)
**All**	**1091**	**1.49 (1.05, 2.11)**	**1501**	**1.70 (1.28, 2.24)**

0.1 0.4 1 4 24

HR (95% CI) per SD higher genetically-predicted adiposity level (men)

0.1 0.4 1 4 24

HR (95% CI) per SD higher genetically-predicted adiposity level (women)

Mendelian randomisation analyses as per Figure 3. *Includes exacerbations of COPD. COPD=Chronic Obstructive Pulmonary Disease.

Figure S7. Associations of genetically-predicted adiposity markers with infection-related mortality at ages 35-74 years in the subset of unrelated participants

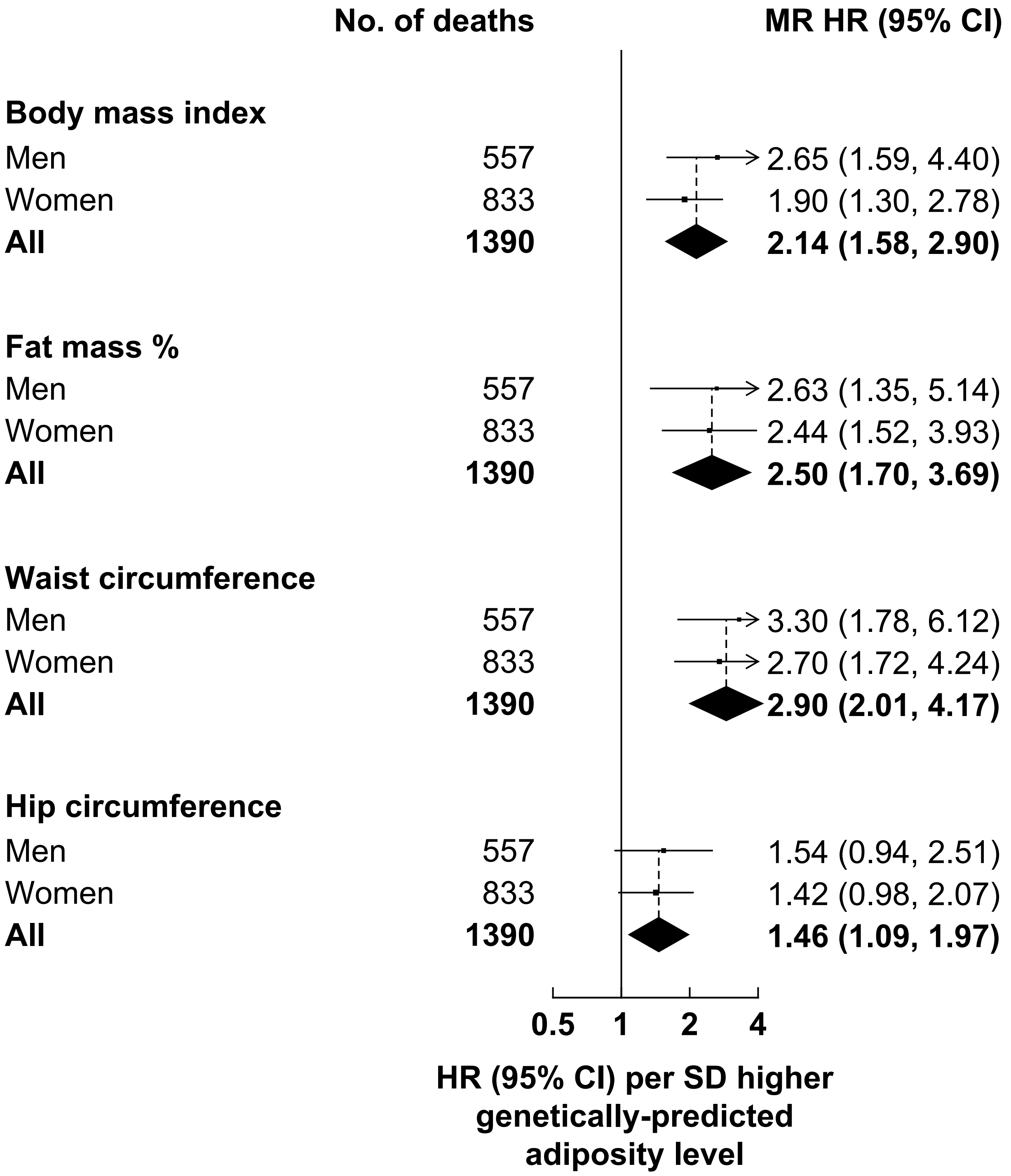

Mendelian randomisation analyses as per Figure 2, but now restricted to participants who are unrelated to the 3rd degree.

Figure S8. Associations of genetically-predicted adiposity markers with infection-related mortality at ages 35-74 years, by selected baseline characteristics

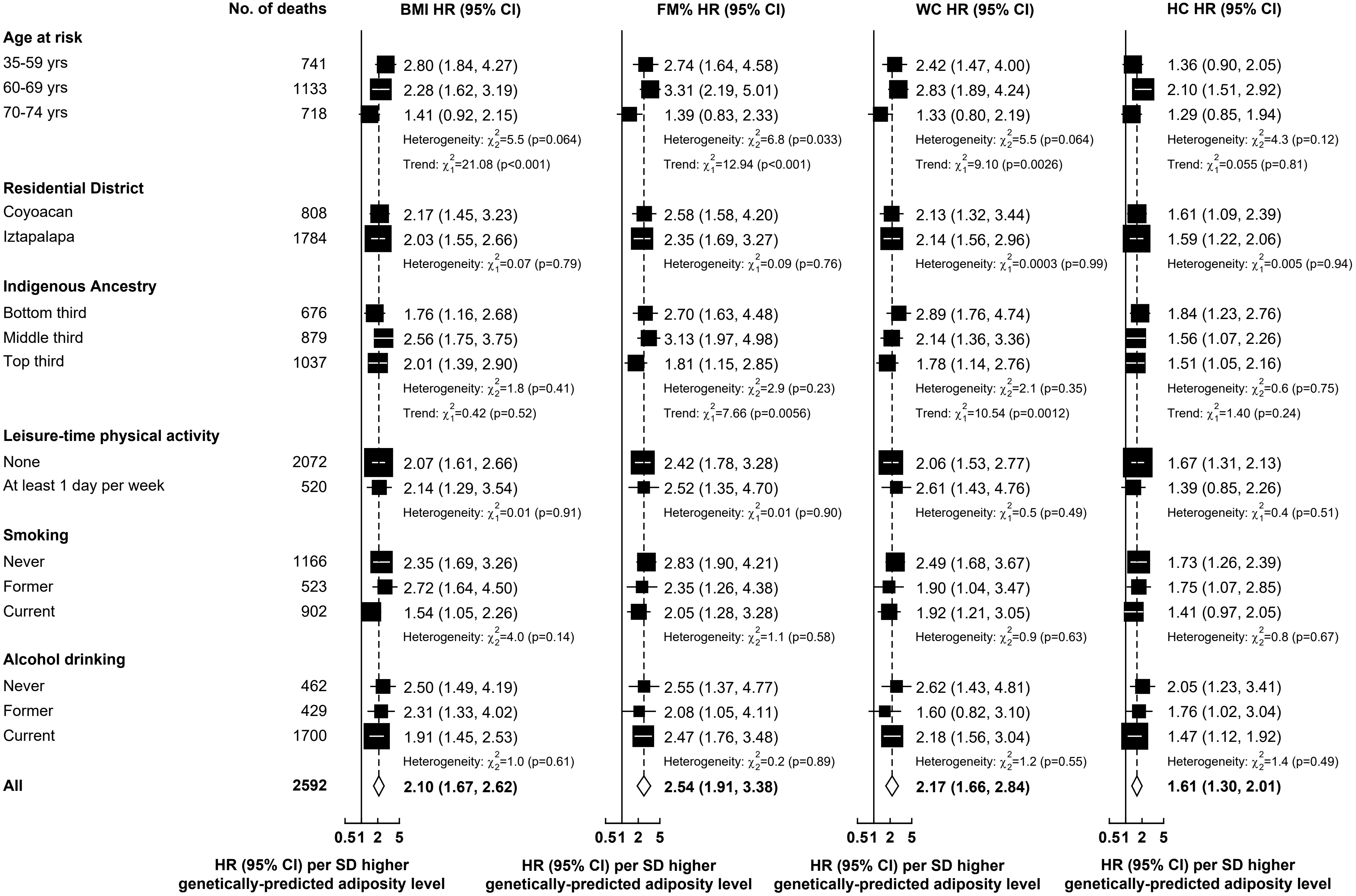

Analyses as per Figure 2 but within particular participant subgroups.

Figure S9. Infection-related mortality across strata of adiposity markers using the residual and doubly−ranked method

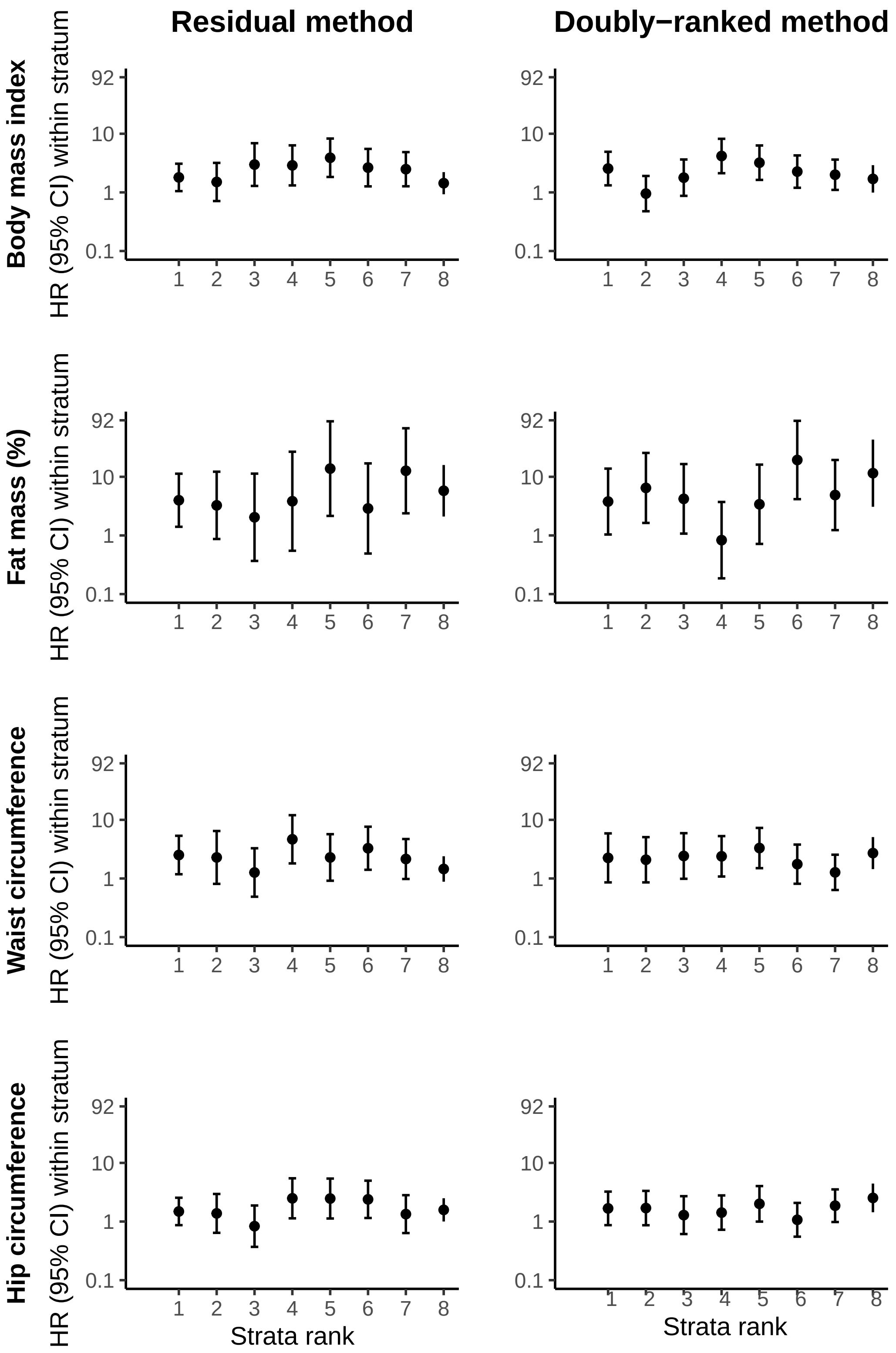

For each adiposity marker (BMI, fat mass %, waist circumference, and hip circumference), the 'residual' method creates 8 equally-sized groups from the residuals after regressing the adiposity marker on its corresponding genetic risk score (GRS). For the 'doubly-ranked' method, participants are first split into 8 equally-sized groups on the basis of the corresponding adiposity marker GRS (the 'first strata'). Then, within each of these 8 groups, participants are split into 8 additional groups on the basis of the corresponding adiposity marker (the 'second strata'). Each of the second strata levels are then combined across levels of the first strata. For example, for BMI, the first group shown above includes those with the lowest BMI in BMI–GRS strata 1, the lowest BMI in BMI–GRS strata 2, and so on, up to and including the lowest BMI in BMI–GRS strata 8. For both methods, the MR ratio method is used to estimate a hazard ratio and 95% CI in each group, in which the adiposity marker GRS to mortality association is estimated only for that group, but the adiposity marker GRS to adiposity marker association is estimated in all participants.

Table S1. ICD-10 classification of infectious disease deaths at ages 35-74 years (genetic analysis population), by underlying cause

Underlying cause	ICD-10 codes (number of deaths at ages 35-74 years)*
All infectious diseases (*n*=2592)	Any of the below
Sepsis (*n*=197) †	A415 (1), A419 (191), A483 (1), R572 (4)
Skin, bone and connective tissue (*n*=126)	A480 (1), L021 (4), L022 (4), L023 (2), L024 (2), L031 (5), L038 (1), L039 (3), L089 (39), L899 (7), L89X (3), L984 (6), M009 (1), M600 (2), M725 (1), M726 (19), M798 (23), M869 (3)
Gastrointestinal infections (*n*=226)	A047 (3), A060 (1), A090 (18), A099 (33), A09X (11), B462 (1), B690 (2), K052 (1), K352 (4), K353 (4), K358 (5), K359 (2), K37X (1), K572 (2), K578 (5), K579 (13), K610 (3), K612 (1), K630 (1), K650 (28), K658 (1), K659 (54), K750 (14), K830 (17), K931 (1)
Urinary tract infections (*n*=227)	N10X (2), N151 (12), N300 (1), N309 (1), N390 (203), N410 (1), N419 (1), N498 (6)
Respiratory infections (*n*=1641)	Any of the below three respiratory subcategories
Pneumonia (*n*=711)	J129 (4), J151 (3), J157 (2), J159 (35), J180 (53), J181 (27), J182 (2), J189 (585)
COVID-19 (*n*=799)	U071 (502), U072 (296), U099 (1)
Bronchitis ‡ (*n*=131)	J209 (4), J22X (17), J42X (14), J440 (94), J441 (2)
Other infections not included above (*n*=175)	A162 (7), A165 (1), A169 (2), A170 (1), A181 (2), A182 (1), A183 (1), A199 (3), A498 (1), A810 (2), A86X (2), B169 (3), B171 (28), B182 (12), B189 (1), B190 (2), B199 (2), B200 (1), B201 (1), B206 (2), B207 (4), B208 (7), B210 (2), B212 (1), B218 (1), B227 (2), B238 (4), B24X (4), B259 (1), B373 (1), B440 (1), B441 (1), B451 (1), B465 (2), B909 (1), B948 (2), B99X (1), G009 (3), G039 (4), G042 (1), G049 (7), G060 (1), H440 (1), H664 (1), H669 (1), I011 (1), I018 (1), I330 (7), I38X (8), J069 (1), J09 (3), J09X (1), J100 (2), J110 (1), J111 (1), J348 (1), J391 (1), J850 (2), J852 (2), J869 (9), N719 (1), N739 (1), T874 (1), Y405 (1)

* Only ICD-10 codes identified as primary causes of death in the study population are presented.

† Without specific source.

‡ Includes exacerbations of chronic obstructive pulmonary disease (COPD).

Table S2: Sex-specific correlations between: a) the genetic instruments for adiposity; and b) the measured levels of adiposity

a) Correlations between genetic instruments (126,491 participants in the genetic analysis population)

	Fat mass %	Body mass index	Waist circumference	Hip circumference	
Fat mass %	1.00	Male			
	1.00	Female			
Body mass index	0.51	1.00	Male		
	0.50	1.00	Female		
Waist circumference	0.57	0.59	1.00	Male	
	0.57	0.59	1.00	Female	
Hip circumference	0.45	0.50	0.52	1.00	Male
	0.45	0.50	0.51	1.00	Female

b) Correlations between measured levels (111,182 participants in the observational analysis population)

	Fat mass %	Body mass index	Waist circumference	Hip circumference	
Fat mass %	1.00	Male			
	1.00	Female			
Body mass index	0.77	1.00	Male		
	0.96	1.00	Female		
Waist circumference	0.97	0.82	1.00	Male	
	0.90	0.82	1.00	Female	
Hip circumference	0.72	0.77	0.80	1.00	Male
	0.85	0.85	0.81	1.00	Female

Fat mass (kg) estimated using Mexican-specific equations from the NHANES (National Health and Nutrition Examination Survey) study (see Supplemental Methods). Fat mass % calculated as 100 multiplied by estimated fat mass divided by weight.

Table S3. Baseline characteristics of 111,182 participants (observational analysis population) aged 35-74 years at recruitment, by sex

	Men	Women	Overall
No. of participants	35,907	75,275	111,182
Age, ancestry and socioeconomic factors			
Age (years)	50 (11)	49 (10)	49 (11)
Indigenous American ancestry, %	0.7 (0.2)	0.7 (0.2)	0.7 (0.2)
Resident of Coyoacán	15,804 (44)	29,196 (39)	45,000 (41)
University or high school educated	9,499 (27)	10,118 (13)	19,617 (18)
Lifestyle factors			
Current smoker	18,752 (52)	18,817 (25)	37,569 (34)
Current alcohol drinker	28,574 (80)	48,907 (65)	77,481 (70)
Physical activity 1+ times/week	11,147 (31)	14,276 (19)	25,423 (23)
Anthropometry, blood pressure and HbA1c			
Height, cm	165 (7)	152 (6)	158 (6.5)
Weight, kg	76 (13)	68 (12)	72 (12)
BMI, kg/m^2	28.0 (4.1)	29.5 (5.1)	28.7 (4.6)
Fat mass, %	28 (4.4)	42 (4.0)	35 (4.2)
Waist circumference, cm	96 (10)	92 (12)	94 (11)
Hip circumference, cm	101 (8)	106 (11)	108 (9)
Blood pressure, mmHg			
Systolic	127 (15)	125 (16)	126 (16)
Diastolic	84 (10)	82 (10)	83 (10)
HbA1c, %	5.5 (0.4)	5.5 (0.4)	5.5 (0.4)

Mean (SD) or n (%). BMI=body mass index. HbA1c=glycosylated haemoglobin A1c. Participants with self-reported diabetes or other chronic disease at baseline were excluded from the observational analysis population.

Table S4. Baseline characteristics of 126,491 participants aged 35-74 years at recruitment (genetic analysis population), by fifth of genetic score for BMI

	Fifth of genetic score for BMI					
	I	II	III	IV	V	Overall
No. of participants	25,299	25,298	25,299	25,298	25,297	126,491
Age, ancestry and socioeconomic factors						
Age (years)	51 (11)	51 (11)	51 (11)	51 (11)	51 (11)	51 (11)
Male	8,166 (32)	8,166 (32)	8,166 (32)	8,166 (32)	8,165 (32)	40,829 (32)
Indigenous American ancestry, %	64 (18)	67 (18)	68 (18)	68 (18)	68 (17)	67 (18)
Resident of Coyoacán	10,180 (40)	9,797 (39)	9,730 (39)	9,369 (37)	9,406 (37)	48,482 (38)
University or high school educated	4,557 (18)	4,079 (16)	3,948 (16)	3,788 (15)	3,709 (15)	20,081 (16)
Lifestyle factors						
Current smoker	7,937 (31)	8,077 (32)	8,274 (33)	8,218 (33)	8,614 (34)	41,120 (33)
Current alcohol drinker	17,291 (68)	17,098 (68)	17,115 (68)	16,961 (67)	16,914 (67)	85,379 (68)
Physical activity 1+ times/week	6,059 (24)	5,752 (23)	5,620 (22)	5,453 (22)	5,413 (21)	28,297 (22)
Anthropometry, blood pressure and HbA1c						
Height (cm)	156 (7)	156 (7)	156 (7)	156 (7)	156 (7)	156 (7)
Weight (kg)	62 (13)	70 (13)	71 (13)	72 (13)	74 (13)	71 (13)
BMI (kg/m^2)	27.9 (4.7)	28.7 (4.7)	29.2 (4.7)	29.6 (4.7)	30.4 (4.7)	29.2 (4.7)
Fat mass (%)	37.2 (4.2)	37.8 (4.2)	38.1 (4.2)	38.4 (4.2)	39.0 (4.2)	38.1 (4.2)
Waist circumference (cm)	92 (11)	93 (11)	94 (11)	95 (11)	97 (11)	94 (11)
Hip circumference (cm)	103 (10)	104 (10)	105 (11)	106 (11)	107 (11)	105 (10)
Blood pressure, mmHg						
Systolic	126.0 (16.3)	126.4 (16.1)	126.8 (16.5)	127.1 (16.3)	127.8 (16.5)	126.8 (16.3)
Diastolic	82.6 (10.2)	82.8 (10.0)	83.0 (10.2)	83.2 (10.1)	83.7 (10.1)	83.1 (10.1)
HbA1c (%)	5.9 (1.6)	6.0 (1.6)	6.1 (1.7)	6.2 (1.8)	6.3 (1.9)	6.1 (1.7)
Medical history						
Diabetes*	3,899 (15%)	4,355 (17%)	4,626 (18%)	5,089 (20%)	5,481 (22%)	23,450 (19%)
Ischaemic heart disease	298 (1.2%)	329 (1.3%)	330 (1.3%)	304 (1.2%)	367 (1.5%)	1,628 (1.3%)
Stroke	197 (0.8%)	230 (0.9%)	223 (0.9%)	235 (0.9%)	278 (1.1%)	1,163 (0.9%)
Other†	2,052 (8.1%)	2,060 (8.1%)	2,033 (8.0%)	2,021 (8.0%)	2,074 (8.2%)	10,240 (8.1%)

Mean (SD) or n (%) shown. BMI=body mass index. HbA1c=glycosylated haemoglobin A1c. Fifths of the genetic instrument distribution are computed separately for men and women; as a result, each fifth contains the same proportion of men by construction.

* Previously diagnosed self-reported diabetes or measured HbA1c ≥6.5%. † Chronic kidney disease, cirrhosis, cancer or emphysema.

Table S5. Baseline characteristics of 126,491 participants aged 35-74 years at recruitment (genetic analysis population), by fifth of genetic score for fat mass percentage

	Fifth of genetic score for fat mass percentage					
	I	II	III	IV	V	Overall
No. of participants	25,299	25,298	25,299	25,298	25,297	126,491
Age, ancestry and socioeconomic factors						
Age (years)	51 (11)	51 (11)	51 (11)	51 (11)	51 (11)	51 (11)
Male	8,166 (32)	8,166 (32)	8,166 (32)	8,166 (32)	8,165 (32)	40,829 (32)
Indigenous American ancestry, %	71 (18)	69 (18)	68 (18)	66 (18)	62 (17)	67 (18)
Resident of Coyoacán	9,432 (37)	9,537 (38)	9,677 (38)	9,802 (39)	10,034 (40)	48,482 (38)
University or high school educated	3,807 (15)	3,919 (16)	4,041 (16)	4,110 (16)	4,204 (17)	20,081 (16)
Lifestyle factors						
Current smoker	7,651 (30)	7,856 (31)	8,223 (33)	8,377 (33)	9,013 (36)	41,120 (33)
Current alcohol drinker	17,050 (67)	17,024 (67)	17,088 (68)	17,151 (69)	17,066 (68)	85,379 (68)
Physical activity 1+ times/week	5,574 (22)	5,627 (22)	5,691 (23)	5,678 (23)	5,727 (23)	28,297 (22)
Anthropometry, blood pressure and HbA1c						
Height (cm)	155 (7)	156 (7)	156 (7)	156 (7)	157 (7)	156 (7)
Weight (kg)	68 (12)	70 (13)	71 (13)	72 (13)	74 (14)	71 (13)
BMI (kg/m^2)	28.3 (4.5)	28.8 (4.8)	29.2 (4.9)	29.5 (5.0)	30.1 (5.3)	29.2 (4.7)
Fat mass (%)	37.4 (4.2)	37.8 (4.2)	38.1 (4.2)	38.4 (4.2)	38.8 (4.2)	38.1 (4.2)
Waist circumference (cm)	92 (11)	93 (11)	94 (11)	95 (12)	97 (12)	94 (11)
Hip circumference (cm)	103 (10)	104 (10)	105 (11)	106 (11)	107 (11)	105 (10)
Blood pressure, mmHg						
Systolic	125.9 (16.2)	126.5 (16.3)	126.9 (16.3)	127.2 (16.4)	127.7 (16.5)	126.8 (16.3)
Diastolic	82.5 (10.1)	82.8 (10.1)	83.1 (10.1)	83.3 (10.2)	83.7 (10.2)	83.1 (10.1)
HbA1c (%)	6.0 (1.6)	6.1 (1.7)	6.1 (1.7)	6.1 (1.7)	6.2 (1.8)	6.1 (1.7)
Medical history						
Diabetes*	4,268 (17%)	4,602 (18%)	4,690 (19%)	4,799 (19%)	5,091 (20%)	23,450 (19%)
Ischaemic heart disease	306 (1.2%)	315 (1.2%)	296 (1.2%)	320 (1.3%)	391 (1.5%)	1,628 (1.3%)
Stroke	196 (0.8%)	215 (0.8%)	218 (0.9%)	274 (1.1%)	260 (1.0%)	1,163 (0.9%)
Other†	1,873 (7.4%)	1,938 (7.7%)	2,055 (8.1%)	2,131 (8.4%)	2,243 (8.9%)	10,240 (8.1%)

Mean (SD) or n (%) shown. BMI=body mass index. HbA1c=glycosylated haemoglobin A1c. Fifths of the genetic instrument distribution are computed separately for men and women; as a result, each fifth contains the same proportion of men by construction.

* Previously diagnosed self-reported diabetes or measured HbA1c ≥6.5%. † Chronic kidney disease, cirrhosis, cancer or emphysema.

Table S6. Baseline characteristics of 126,491 participants aged 35-74 years at recruitment (genetic analysis population), by fifth of genetic score for waist circumference

	Fifth of genetic score for waist circumference					
	**I**	**II**	**III**	**IV**	**V**	**Overall**
No. of participants	25,299	25,298	25,299	25,298	25,297	126,491
Age, ancestry and socioeconomic factors						
Age (years)	51 (11)	51 (11)	51 (11)	51 (11)	51 (11)	51 (11)
Male	8,166 (32)	8,166 (32)	8,166 (32)	8,166 (32)	8,165 (32)	40,829 (32)
Indigenous American ancestry, %	73 (17)	70 (017)	68 (17)	65 (17)	60 (17)	67 (18)
Resident of Coyoacán	9,276 (37)	9,484 (38)	9,705 (38)	9,766 (39)	10,251 (41)	48,482 (38)
University or high school educated	3,744 (15)	3,850 (15)	3,996 (16)	4,038 (16)	4,453 (18)	20,081 (16)
Lifestyle factors						
Current smoker	7,364 (29)	7,807 (31)	8,201 (32)	8,520 (34)	9,228 (37)	41,120 (33)
Current alcohol drinker	16,877 (67)	17,063 (68)	17,199 (68)	17,127 (68)	17,113 (68)	85,379 (68)
Physical activity 1+ times/week	5,581 (22)	5,572 (22)	5,561 (22)	5,778 (23)	5,805 (23)	28,297 (22)
Anthropometry, blood pressure and HbA1c						
Height (cm)	155 (7)	155 (7)	156 (7)	156 (7)	157 (7)	156 (7)
Weight (kg)	67 (13)	70 (13)	71 (13)	72 (13)	74 (14)	71 (13)
BMI (kg/m^2)	28.2 (4.6)	28.9 (4.8)	29.1 (4.9)	29.5 (5.0)	30.1 (5.3)	29.2 (4.7)
Fat mass (%)	37.4 (4.2)	37.9 (4.2)	38.1 (4.2)	38.3 (4.2)	38.7 (4.2)	38.1 (4.2)
Waist circumference (cm)	92 (11)	94 (11)	94 (11)	95 (12)	97 (12)	94 (11)
Hip circumference (cm)	103 (10)	104 (10)	105 (11)	105 (11)	107 (11)	105 (10)
Blood pressure, mmHg						
Systolic	126.0 (16.2)	126.6 (16.2)	126.8 (16.3)	127.1 (16.5)	127.7 (16.5)	126.8 (16.3)
Diastolic	82.5 (10.1)	82.9 (10.1)	83.0 (10.2)	83.2 (10.1)	83.7 (10.2)	83.1 (10.1)
HbA1c (%)	6.0 (1.6)	6.1 (1.7)	6.1 (1.7)	6.1 (1.8)	6.2 (1.8)	6.1 (1.7)
Medical history						
Diabetes*	4,234 (17%)	4,517 (18%)	4,637 (18%)	4,913 (19%)	5,149 (20%)	23,450 (19%)
Ischaemic heart disease	292 (1.2%)	288 (1.1%)	341 (1.3%)	321 (1.3%)	386 (1.5%)	1,628 (1.3%)
Stroke	211 (0.8%)	217 (0.9%)	254 (1.0%)	236 (0.9%)	245 (1.0%)	1,163 (0.9%)
Other†	1,882 (7.4%)	2,007 (7.9%)	1,974 (7.8%)	2,065 (8.2%)	2,312 (9.1%)	10,240 (8.1%)

Mean (SD) or n (%) shown. BMI=body mass index. HbA1c=glycosylated haemoglobin A1c. Fifths of the genetic instrument distribution are computed separately for men and women; as a result, each fifth contains the same proportion of men by construction.

* Previously diagnosed self-reported diabetes or measured HbA1c ≥6.5%. † Chronic kidney disease, cirrhosis, cancer or emphysema.

Table S7. Baseline characteristics of 126,491 participants aged 35-74 years at recruitment (genetic analysis population), by fifth of genetic score for hip circumference

	Fifth of genetic score for hip circumference					
	**I**	**II**	**III**	**IV**	**V**	**Overall**
No. of participants	25,299	25,298	25,299	25,298	25,297	126,491
Age, ancestry and socioeconomic factors						
Age (years)	51 (11)	51 (11)	51 (11)	51 (11)	51 (11)	51 (11)
Male	8,166 (32)	8,166 (32)	8,166 (32)	8,166 (32)	8,165 (32)	40,829 (32)
Indigenous American ancestry, %	68 (18)	68 (18)	68 (18)	67 (18)	65 (18)	67 (18)
Resident of Coyoacán	9,773 (39)	9,711 (38)	9,735 (39)	9,514 (38)	9,749 (39)	48,482 (38)
University or high school educated	4,025 (16)	3,980 (16)	3,960 (16)	3,985 (16)	4,131 (16)	20,081 (16)
Lifestyle factors						
Current smoker	7,794 (31)	8,016 (32)	8,231 (33)	8,335 (33)	8,744 (35)	41,120 (33)
Current alcohol drinker	17,130 (68)	17,083 (68)	16,998 (67)	17,044 (67)	17,124 (68)	85,379 (68)
Physical activity 1+ times/week	5,792 (23)	5,575 (22)	5,590 (22)	5,624 (22)	5,716 (23)	28,297 (22)
Anthropometry, blood pressure and HbA1c						
Height (cm)	155 (7)	155 (7)	156 (7)	156 (7)	157 (7)	156 (7)
Weight (kg)	67 (13)	70 (13)	71 (13)	72 (13)	75 (13)	71 (13)
BMI (kg/m^2)	28.1 (4.5)	28.7 (4.8)	29.1 (4.9)	29.5 (5.0)	30.3 (5.3)	29.2 (4.7)
Fat mass (%)	37.3 (4.2)	37.7 (4.2)	38.2 (4.2)	38.4 (4.2)	38.9 (4.2)	38.1 (4.2)
Waist circumference (cm)	92 (11)	93 (11)	94 (11)	95 (11)	97 (11)	94 (11)
Hip circumference (cm)	102 (10)	104 (10)	105 (10)	106 (11)	108 (11)	105 (10)
Blood pressure, mmHg						
Systolic	126.3 (16.2)	126.6 (16.3)	126.7 (16.5)	127.1 (16.4)	127.5 (16.3)	126.8 (16.3)
Diastolic	82.7 (10.1)	82.9 (10.1)	83.0 (10.2)	83.3 (10.2)	83.5 (10.1)	83.1 (10.1)
HbA1c (%)	6.0 (1.7)	6.1 (1.7)	6.1 (1.7)	6.1 (1.8)	6.2 (1.8)	6.1 (1.7)
Medical history						
Diabetes*	4,424 (18%)	4,507 (18%)	4,662 (18%)	4,830 (19%)	5,027 (20%)	23,450 (19%)
Ischaemic heart disease	317 (1.3%)	305 (1.2%)	353 (1.4%)	324 (1.3%)	329 (1.3%)	1,628 (1.3%)
Stroke	231 (0.9%)	200 (0.8%)	245 (1.0%)	240 (0.9%)	247 (1.0%)	1,163 (0.9%)
Other†	1,886 (7.5%)	1,976 (7.8%)	2,046 (8.1%)	2,067 (8.2%)	2,265 (9.0%)	10,240 (8.1%)

Mean (SD) or n (%) shown. BMI=body mass index. HbA1c=glycosylated haemoglobin A1c. Fifths of the genetic instrument distribution are computed separately for men and women; as a result, each fifth contains the same proportion of men by construction.

* Previously diagnosed self-reported diabetes or measured HbA1c ≥6.5%. † Chronic kidney disease, cirrhosis, cancer or emphysema.

Table S8. Sensitivity 'two-sample' MR analyses for death from infection at ages 35-74 years, using SNP-to-adiposity associations derived from a trans-ancestry GWAS of China Kadoorie Biobank and UK Biobank

	Mortality HR (95% CI) per SD higher genetically-predicted adiposity marker							
	**Body mass index**		**Fat mass (%)**		**Waist circumference**		**Hip circumference**	
	**Men**	**Women**	**Men**	**Women**	**Men**	**Women**	**Men**	**Women**
Inverse-variance weighted (IVW)[a]	1.65 (1.30-2.08)	1.61 (1.36-1.89)	1.31 (1.09-1.57)	1.51 (1.32-1.75)	1.40 (1.08-1.83)	1.90 (1.49-2.41)	1.36 (1.05-1.76)	1.61 (1.26-2.03)
MR-Egger[b]	1.53 (0.77-3.07)	1.15 (0.71-1.87)	1.60 (0.86-2.94)	1.67 (1.03-2.69)	0.82 (0.36-1.88)	1.13 (0.52-2.41)	1.38 (0.65-2.93)	2.25 (1.12-4.56)
Weighted median[c]	1.65 (1.13-2.40)	1.47 (1.11-1.93)	1.20 (0.91-1.59)	1.44 (1.15-1.80)	1.10 (0.72-1.65)	1.79 (1.19-2.66)	1.45 (0.96-2.18)	1.70 (1.16-2.50)
MR-PRESSO[d]	1.65 (1.30-2.08)	1.56 (1.32-1.84)	1.31 (1.09-1.57)	1.51 (1.32-1.75)	1.40 (1.08-1.83)	1.82 (1.42-2.32)	1.36 (1.05-1.76)	1.55 (1.22-1.96)

HR (95% CI) shown per 1 SD higher genetically-predicted adiposity marker (BMI, fat mass (%), waist circumference, and hip circumference) using various 'two sample' MR approaches (all adjusted for age and the first 7 genetic principal components). For each approach, the SNP-adiposity marker effect estimates and their standard errors are taken from trans-ancestry meta-analysis of China Kadoorie Biobank with UK Biobank, while the SNP-mortality effect estimates and their standard errors are taken from MCPS. For all approaches, the SNPs are aligned so that the adiposity-specific SNP-trait associations are strictly positive. Details of the four methods can be found in https://cran.r-project.org/web/packages/MendelianRandomization/MendelianRandomization.pdf. Brief details and underlying assumptions are provided below.

[a]An inverse variance weighted (IVW) linear regression of the SNP-mortality effects on the (aligned) SNP-adiposity effects is performed in which the regression line is forced through the origin. It relies on the assumption of no 'horizontal pleiotropy' (i.e., it assumed that all of the SNPs affect mortality only through the adiposity trait and not through any other mechanism).

[b]A modifications of the IVW approach in which the regression is not forced through the origin. The intercept provides an estimate of net horizontal pleiotropic effects under the assumption that any such effects are uncorrelated with the SNP-exposure effects (known as the InSIDE assumption). For each HR shown in the above table, the intercept term was non-significant at the 5% level. The estimated intercepts were 0.001 for BMI in men and 0.007 in women; −0.005 for FM% in men and −0.002 in women; 0.010 for WC in men and 0.009 in women; and −0.0002 for HC in men and -0.0052 in women, respectively.

[c]The weighted median approach applies the ratio method to each SNP, orders the results, assigns them normalised inverse-variance weights (to capture the 'information content' of each SNP), then selects the ratio estimate at which the cumulative information content first passes half of the total information content. It relies on the assumption that at least half of the statistical information on the SNP-BMI associations comes from valid instrumental variables. It is more robust to individual genetic variants with strongly outlying causal estimates compared with the IVW and MR-Egger methods.

[d]Outlier-corrected IVW (MR-PRESSO) applies the IVW approach but to a reduced set of SNPs in which any SNPs contributing significant heterogeneity (after Bonferroni-correction) to the estimated IVW slope are removed, and then the IVW approach is re-applied to the remaining SNPs.

Supplementary methods

Study design and participants

Details of the MCPS design, methods and participants have been described previously.[w1] Between 1998 and 2004 households in two urban districts of Mexico City (Coyoacán and Iztapalapa) were visited and residents aged 35 or older were invited to participate in the study. Of the 112,333 households with eligible residents, at least one individual from 106,059 (94%) households participated. Of the 159,755 participants recruited, about two thirds were women and one third men, reflecting that most home visits were carried out during working hours, when women were more likely to be at home. Ethics approval for the study was obtained from the Mexican Ministry of Health, the Mexican National Council for Science and Technology, and the University of Oxford, UK. All participants provided written informed consent.

Data collection

During household visits trained nurses administered electronic questionnaires recording sociodemographic and lifestyle factors, current medications, and medical history. Calibrated electronic scales, stadiometers, and non-stretchable tape were used to measure weight (to the nearest 0.1 kg), height (to the nearest 0.1 cm), and waist and hip circumference (also to the nearest 0.1cm), respectively. Body mass index was calculated as weight in kilograms divided by the square of height in metres. Total fat mass (in kilograms) was predicted based on age, sex, height, weight, and waist circumference through the following Mexican-specific equations derived from the NHANES study:[w2]

Fat mass in men = –18.592 – (0.009 x age in years) – (0.080 x height in cms) + (0.226 x weight in kgs) + (0.387 x waist circumference in cms) + 0.08 (specific term for Mexican population)

Fat mass in women = 11.817 + (0.041 x age in years) – (0.199 x height in cms) + (0.61 x weight in kgs) + (0.044 x waist circumference in cms) + 0.388 (specific term for Mexican population)

These predicted levels of fat mass were validated in a subset of participants with direct bioimpedance measures.[w3] Fat mass percentage (FM%) was then calculated as 100 multiplied by the predicted fat mass divided by total weight.

Blood pressure was measured with the participant seated. A 10-mL non-fasting venous blood sample was collected into an EDTA vacutainer, separated into plasma and buffy coat aliquots and stored long-term at -150°C. Glycosylated haemoglobin (HbA1c) was measured in buffy coat samples using validated high-performance liquid chromatography[w4] on HA-8180 analysers with calibrators traceable to International Federation of Clinical Chemistry standards.[w5] Previously-diagnosed diabetes was defined as a self-reported doctor diagnosis or regular use of a diabetes medication, while undiagnosed diabetes was defined as a baseline HbA1c concentration ≥6.5% (≥48 mmol/mol) in someone without previously-diagnosed diabetes. Genotyping was performed in all participants with version 2 of the Illumina Global Screening Array.[w6] Genetic imputation, and quality control procedures including individual-level missingness, departures from Hardy-Weinberg equilibrium, and Mendel errors were conducted as previously described.[w7] Per-individual proportions of Indigenous, European, African and East Asian inherited ancestry (i.e., the different proportions of their genomes descended from previously discrete subpopulations), were estimated.[w7]

Exclusion criteria

All analyses excluded those aged ≥75 years at recruitment, those with uncertain mortality linkage (defined as a ≥1 year one discrepancy in a participant's date of birth as recorded at the baseline survey compared with the matched death certificate), and duplicate assessments of the few participants who were recruited twice (data from the first visit at which they provided a blood sample were retained). The Mendelian randomisation analyses further excluded those with missing genetic data or genetic data that did not pass quality control.[w7] The observational analyses excluded those with missing covariate data, those with anthropometric measurements that were considered to be outliers (height <120 or >200 cms , weight <35 or >250 kgs, waist circumference <60 or >180 cm, hip

circumference <70 or >180 cm, waist-to-hip ratio <0.5 or >1.5, or BMI <15 or >60 kg/m^2), and those with self-reported previously-diagnosed chronic diseases (diabetes, ischaemic heart disease, stroke, cancer, chronic kidney disease, cirrhosis, or emphysema), in order to reduce reverse causality bias.

Instrumental variables for adiposity markers

The main analyses used a genetic instrument score (GS) for each adiposity marker (BMI, Fat Mass % [FM %], waist and hip circumferences [HC]), using results from a trans-ancestry meta-analysis of results from genome-wide association studies of a large East Asian and a large European population.[w8] Specifically, 907, 768, 633, and 803 conditionally-independent genetic variants associated with BMI, FM %, waist circumference and HC, respectively, at genome-wide significance level ($P \leq 5x10^{-8}$) and LD-clumped at an r^2 threshold of 0.05 were initially selected from the trans-ancestry meta-analysis. After excluding unavailable or ambiguous variants in the MCPS genetic imputed data (as direct matches or proxies based on a linkage disequilibrium [LD] at an $r^2 \geq 0.8$, or the allelic strand could not be harmonized), the remaining variants included 781 SNPs for BMI, 675 SNPs for FMP, 547 SNPs for WC, and 707 SNPs for HC. After further clumping of variants in linkage-disequilibrium (based on $r^2 \geq 0.001$ and a clumping window of ±5 Mb) the final set of SNPs for inclusion in the trans-ancestry polygenic instrument included 724 variants for BMI, 636 for FM%, 521 for waist circumference, and 651 for HC. Each participant's adiposity-specific genetic score was calculated as the linear combination of the SNP-dosages multiplied by their weights (which were the same irrespective of sex for the trans-ancestry GS).

Main regression models and covariate adjustment

The Wald ratio method[w9] was used to assess the causal relevance of each adiposity marker to cause-specific mortality. For this ratio, Cox regression was used to estimate the sex-specific association between the relevant trans-ancestry GS and mortality, with adjustment for age-at-risk (in 5-year-bands) and the first seven genetic principal components (PCs). Adjustment for age-at-risk rather than baseline age was done to provide a more complete adjustment for the potential confounding effects of age (as well as for consistency with previous epidemiological analyses of BMI and mortality).[w10] For the denominator of the Wald ratio, linear regression estimated the sex-specific association between the relevant trans-ancestry GS and the adiposity marker, with adjustment for age, age-squared and the first seven genetic PCs. Seven PCs were chosen (rather than, for example, ten) because higher PCs in the MCPS population display localized genomic regions with increased loadings due to long-range linkage-disequilibrium.[w7] In sensitivity analyses we performed a range of additional 'two-sample' MR approaches (inverse variance weighted, MR-Egger, weighted median and outlier corrected inverse variance weighted [MR-PRESSO]).[w9] These analyses used the SUGEN software[w12] (which accounts for relatedness) to estimate the mortality log hazard ratio associated with each variant. A description of these 2-sample MR approaches and their assumptions is provided in Web**Table S8**.

Observational analyses

In observational analyses, Cox regression models, with age-at-risk as the underlying timescale, were used to assess the relevance of each adiposity marker measured at recruitment with death from infection at ages 35-74 years. Hazard ratios were estimated across five-equally-sized groups of the sex-specific distributions of each adiposity marker (to assess the approximate shape of the associations), as well as per one standard deviation (SD) higher level of each adiposity marker (to assess the average strength of association across the adiposity distribution), where the SD was used was the average of the sex-specific SDs. Observational models were adjusted for age-at-risk, sex, district of residence (Coyoacán, Iztapalapa), education attained (university/high school, elementary school, other), leisure-time physical activity (none, up to twice weekly, at least three times weekly), smoking status (never, former, current) and alcohol drinking (never, former, current). To reduce potential reverse causality in these observational analyses, particularly weight loss due to poorly controlled diabetes, these analyses were restricted to the subset of participants without diabetes or other chronic disease at baseline.[w10] However, it should be noted that this restriction also largely removes the effects of adiposity on infection that may be mediated *through* diabetes, and are therefore

may underestimate the total impact of adiposity on infection in the overall population, limiting direct comparability with the MR analyses.

Mortality follow-up

Death registration in Mexico City is reliable and complete, with almost all deaths certified medically.[w13] Participants are followed for cause-specific mortality through probabilistic linkage (based on name, including phonetic coding of names, age, and sex) to the Mexican System for Epidemiologic Death Statistics (*Subsistema Epidemiológico y Estadístico de Defunciones* or SEED) electronic death registry in Mexico City, administered by the Ministry of Health. Field-worker follow-up of more than 7000 deaths matched in this way confirmed that the match was correct in more than 95% of cases. Diseases recorded on death certificates are coded using the International Statistical Classification of Diseases and Related Health Problems, Tenth Revision, with subsequent review by study clinicians to recode, when necessary, the underlying cause of death.[w15] Participant deaths were tracked from recruitment (1998 to 2004) until October 1, 2022.

Assessment of the assumptions underlying Mendelian randomisation

The assumptions of Mendelian randomisation are that: (i) the instrument is associated with the exposure ("relevance"); (ii) the instrument-to-outcome association is not confounded ("independence"); and (iii) the genetic instrument affects the outcome only through the exposure of interest ("exclusion restriction").[w16] The first assumption was assessed by using analysis of variance to estimate the percent of variance in observed adiposity markers explained by the adiposity-GS, and also by plotting the mean of each adiposity marker across fifths of each corresponding GS. The second assumption was assessed by adjusting for genetic principal components to account for population stratification, and by tabulating mean levels of potential confounders across fifths of each adiposity-specific GS. To explore potential violations of the third assumption, additional sensitivity analyses using two-sample MR were conducted. For the second and third assumptions, these investigations are meant to provide supportive, but not definitive, evidence regarding these assumptions.

Appendix references

w1. Tapia-Conyer R, Kuri-Morales P, Alegre-Díaz J, et al. Cohort profile: the Mexico City Prospective Study. Int J Epidemiol 2006; 35(2): 243-9

w2. Lee DH, Keum N, Hu FB, Orav EJ, Rimm EB, Sun Q, Willett WC, Giovannucci EL. Development and validation of anthropometric prediction equations for lean body mass, fat mass and percent fat in adults using the National Health and Nutrition Examination Survey (NHANES) 1999-2006. Br J Nutr. 2017;118:858–866.

w3. Gnatiuc Friedrichs L, Wade R, Alegre-Díaz J, et al. Body Composition and Risk of Vascular-Metabolic Mortality Risk in 113 000 Mexican Men and Women Without Prior Chronic Disease. *Journal of the American Heart Association* 2023; **12**(3): e028263.

w4. Youngman LD, Clark S, Manley S, Peto R, Collins R. Reliable measurement of glycated hemoglobin in frozen blood samples: implications for epidemiologic studies. Clin Chem 2002; 48:1627-9.

w5. International Federation of Clinical Chemistry. Standardization of HbA1c. NGSP (online http://www.ngsp.org/docs/IFCCstd.pdf).

w6. Illumina Infinitum Global Screening Array (https://www.illumina.com/content /dam/illumina-marketing/documents/products/datasheets/infinium-global-screening-array-data-sheet-370-2016-016.pdf)

w7. Ziyatdinov A, Torres J, Alegre-Díaz J, et al. Genotyping, sequencing and analysis of 140,000 adults from Mexico City. Nature 2023; 622: 784-93

w8. Fairhurst-Hunter Z, Lin K, Millwood IY, Pozarickij A, Chen T-T, Torres JM, et al. Trans-ancestry meta-analysis improves performance of genetic scores for multiple adiposity-related traits in East Asian populations. MedRxiv. 2022:2022.07.05.22277254.

w9. Burgess S, Thompson SG: Mendelian Randomization: Methods for using Genetic Variants in Causal Estimation. Taylor & Francis Group; 2015
w10. Gnatiuc L, Alegre-Diaz J, Wade R, Ramirez-Reyes R, Tapia-Conyer R, Garcilazo-Avila A, et al. General and Abdominal Adiposity and Mortality in Mexico City: A Prospective Study of 150 000 Adults. Ann Int Med. 2019;171:397-405
w11. Burgess S, Bowden J, Fall T, Ingelsson E, Thompson SG. Sensitivity Analyses for Robust Causal Inference from Mendelian Randomization Analyses with Multiple Genetic Variants. Epidemiology (Cambridge, Mass). 2017;28:30-42
w12. Lin D-Y, Tao R, Kalsbeek WD, Zeng D, Gonzalez F, Fernández-Rhodes L, et al. Genetic association analysis under complex survey sampling: the Hispanic Community Health Study/Study of Latinos. Am J Hum Gen. 2014; 95: 675-88
w13. Mikkelsen L, Phillips DE, AbouZahr C, et al. A global assessment of civil registration and vital statistics systems: monitoring data quality and progress. Lancet 2015; 386: 1395-406.
w14. Alegre-Diaz J, Herrington W, Lopez-Cervantes M, et al. Diabetes and Cause-Specific Mortality in Mexico City. NEJM 2016; 375: 1961-71.
w15. International Statistical Classification of Diseases and Related Health Problems: 10th revision (ICD-10). 2. Geneva: World Health Organisation, 2004.
w16. Skrivankova VW, Richmond RC, Woolf BAR et al. Strengthening the Reporting of Observational Studies in Epidemiology Using Mendelian Randomization: The STROBE-MR Statement. JAMA 2021; 326: 1614-1621.
